# Isolation of bacteriophages infecting *Xanthomonas oryzae* pv. *oryzae* and genomic characterization of novel phage vB_XooS_NR08 for biocontrol of bacterial leaf blight of rice

**DOI:** 10.3389/fmicb.2023.1084025

**Published:** 2023-03-16

**Authors:** Lata Jain, Vinay Kumar, Sanjay Kumar Jain, Pankaj Kaushal, Probir Kumar Ghosh

**Affiliations:** ICAR-National Institute of Biotic Stress Management, Raipur, Chhattisgarh, India

**Keywords:** bacteriophages, *Xanthomonas oryzae* pv. *oryzae*, NR08 phage, bacterial leaf blight, whole-genome sequencing, rice, biocontrol

## Abstract

Bacterial leaf blight (BLB) disease of rice caused by *Xanthomonas oryzae* pv*. oryzae* (*Xoo*) is one of the most destructive diseases worldwide in rice-growing regions. The Ineffectiveness of chemicals in disease management has increased the interest in phage therapy. In this study, we isolated 19 bacteriophages, infecting *Xoo*, from a rice field, which belonged to phage families *Siphoviridae, Myoviridae*, and *Podoviridae* on the basis of electron microscopy. Among 19 phages, Phage vB_XooS_NR08, a member of the *Siphoviridae* family, expressed antibacterial activity against all *Xoo* strains tested and did not lyse *X. campestris* and other unrelated bacterial hosts. Phage NR08 showed more than 80% viability at a temperature range of 4°C–40°C, pH range of 5–9, and direct exposure to sunlight for 2 h, whereas UV light and chemical agents were highly detrimental. In a one-step growth curve, NR08 has a 40-min latent period, followed by a 30-min burst period with a burst size of 250 particle/bacterium. The genome of NR08 is double-stranded DNA, linear having a size of 98,812 bp with a G + C content of 52.9%. Annotation of the whole-genome sequence indicated that NR08 encodes 142 putative open reading frames (ORFs), including one ORF for tRNA, namely, trna1-GlnTTG. Comparative genome analysis of NR08 showed that it shares maximum similarity with *Pseudomonas* phage PaMx42 (40% query coverage, 95.39% identity, and acc. Length 43,225) and *Xanthomonas* phage Samson (40% query coverage, 96.68% identity, and acc. Length 43,314). The average alignment percentage (AP) of NR08 with other Xoophages was only 0.32 to 1.25 since the genome of NR08 (98.8 kb) is almost double of most of the previously reported Xoophages (43–47 kb), thus indicating NR08 a novel Xoophage. In *in vitro* bacterial challenge assay, NR08 showed bacteriostasis up to 24 h and a 99.95% reduction in bacterial growth in 48 h. In rice pot efficacy trials, single-dose treatment of NR08 showed a significant reduction in disease up to 90.23% and 79.27% on 7 and 21 dpi, respectively. However, treatment using 2% skim milk-supplemented phage preparation was significantly less effective as compared to the neat phage preparation. In summary, this study characterized a novel Xoophage having the potential as a biocontrol agent in the mitigation of BLB in rice.

## Introduction

Rice is a staple crop worldwide, particularly in Asian countries with nearly 90% of total global production and consumption contributing immensely to food and nutritional security urging essentiality for sustainable rice production globally (Fukagawa and Ziska, 2019). Numerous biotic stresses emerged as major constraints for rice cultivation, among which bacterial blight (BLB) of rice is one of the most destructive diseases of rice in Asia (Mew, 1987). It is caused by *Xanthomonas oryzae* pv*. oryzae* (*Xoo*), a Gram-negative bacteria that belong to the family *Xanthomonadaceae* subclass Gammaproteobacteria (Nakayinga et al., 2021). BLB infection leads to yield loss of 20%–70% depending on the variety and severity of infection, especially in hybrid rice, the stage of the crop, and climatic conditions, and further, the degree of yield loss was affected by the stage of BLB infection (Khush et al., 1989; Niño-Liu et al., 2006; Chukwu et al., 2019). The losses may be even 80%–100% in case of bacterial infection occurring at the tillering stage of the crop (Srinivasan and Gnanamanickam, 2005; Khan et al., 2012). BLB affects photosynthetic areas and drastically reduces the yield, producing partially filled grain and low-quality fodder yield (Pradhan et al., 2015). In India, this disease is prevalent in almost all paddy growing regions and states and is a major problem in the Kharif season crop (Laha et al., 2009).

*Xoo* pathogen produces two distinct phases of the disease: (i) the leaf blight phase affecting mature plants in which bacteria enter through hydathodes present in the leaf tip and leaf margin, multiply in the intercellular spaces, and then migrate to the xylem vessels, cause infection, and result in yellow to tannish-gray to white lesions along the veins with irregular wavy margins; and (ii) the Kresek phase is systemic infection during seedling stage and results in desiccation of leaves and death of young transplanted plants (Ou, 1985; Banerjee et al., 2018).

The conventional methods recommended for managing BLB disease include cultural practices, chemicals, antibiotics, and biological control agents which remain ineffective, particularly in the epidemic form of the disease (Kumar et al., 2020). Thus, disease management is difficult nowadays due to the lack of effective bactericides and chemicals; high pathogen variability; races; gene transfers in pathogens leading to the development of antibiotics resistance; and development of bacterial resistance to copper (Obradovic et al., 2004). Therefore, the search for environment-friendly alternatives to chemical anti-bacterials has led to the re-evaluation of phage therapy, an alternative strategy for the control of phytopathogens using naturally occurring bacteriophages.

Phages are naturally occurring viruses and are the most abundant entities on earth that specifically infect their host bacteria and have no direct negative effects on plants, animals, or humans. Infection of a host bacterium by a virulent phage leads to rapid viral replication inside the bacterial cell followed by its lysis and killing and finally release of numerous progeny phages (Svircev et al., 2018). Phages are self-replicating and self-limiting; thus, they reproduce only as long as their host bacterium is present in the environment and quickly degrade in absence of the host. They are nontoxic to eukaryotic cells and do not destroy other beneficial microbes in the normal microbiota; hence, they are highly host-specific. Their application has minimal or no side effects. Thus, bacteriophages offer an alternative eco-friendly control measure over conventional management strategies for controlling such bacterial diseases in the plant (Balogh et al., 2008; Addy et al., 2012). The therapeutic use of bacteriophages to treat pathogenic bacterial infections is known as phage therapy.

In recent years, the phage therapy approach for the control of bacterial pathogens has become highly attractive with the proven reports of the efficacy of phage in the control of bacterial hosts (Svircev et al., 2018). In agriculture, bacteriophages have been isolated and/or phage therapy research has been explored as an effective tool to control several phytopathogenic bacteria, including *Xanthomonas* spp. (bacterial spot of peach; Ceverolo and Keil, 1969), *X. campestris* pv*. vesicatoria* (Balogh et al., 2003), *Pectobacterium carotovorum* (Ravensdale et al., 2007), *Pseudomonas* spp. (bacterial blotch of mushroom; Kim et al., 2011), *Ralstonia* spp. (bacterial wilt of tobacco; Fujiwara et al., 2011), *Dickeya* spp. (blackleg disease of potato; Adriaenssens et al., 2012), *Pectobacterium* spp. (blackleg disease of potato; Lim et al., 2013), *Xanthomonas oryzae* pv. *oryzae* (BLB of rice; Chae et al., 2014), *Dickeya solani* (soft rot *Enterobacteriaceae*; Carstens et al., 2018), and *Xanthomonas axonopodis* pv*. allii* (bacterial leaf blight of welsh onions; Nga et al., 2021). The use of bacteriophages against plant pathogenic bacteria as biopesticides has opened new opportunities for the control of devastating bacterial diseases, against which no effective measures exist in the present era of antimicrobial resistance.

This study focused on the isolation of phages against rice bacterial blight pathogen *Xanthomonas oryzae* pv. *oryzae*, and effect of environmental factors on the phage stability and whole-genome sequencing of potential and novel phage vB_XooS_NR08 and its efficacy to control BLB disease in the rice pot.

## Materials and methods

### Studied area

This study was carried out at ICAR-National Institute of Biotic Stress Management (NIBSM), Raipur, Chhattisgarh, India. BLB-infected samples were collected from five states of India, *viz.*, Chhattisgarh (Raipur 21.24 N 81.633 E), Odisha (Cuttack 20.46 N 85.8792 E), Meghalaya (Umiam 25.676 N 91.927 E), Tripura (Agartala 23.831 N 91.287 E), and Assam (Guwahati 26.7269 N 94.2038 E), whereas rice field samples for bacteriophage isolation were collected from all districts of Chhattisgarh (Latitude 17°46′ N to 24°5′ North and Longitude 80°15′ E to 84°20′ East) and adjoining states. Further details of sampling locations of isolated phages are mentioned in Table 1.

**Table 1:** Table showing the details of isolation source and sites, plaque size, and host range of 19 bacteriophages against *Xanthomonas oryzae* pv*. oryzae.*

Phage name	Place of sampling site (latitude and longitude)	Isolation source	Avg. plaque size in mm and plaque type	Plaque count (PFU/mL)	Infectivity (lysis of bacterial cell) to *Xanthomonas oryzae* pv*. oryzae* isolates						Infectivity to heterologous host *viz*., *Bacillus* spp., *Enterobacter* spp., *Pseudomonas* spp. etc
					XooCG01	XooCG02	XooCG03	XooOD01	XooOD02	XooTR01	
NR01	Raipur, CG (21.552788 N 81.784172 E)	Rice field Water	2–3 mm, Clear	3.8 × 10^7^	+	+	+	+	+	−	−
NR02	Rajnandgaon, CG (21.0971114 N 81.0302142 E)	Rice field Water	3–4 mm, Clear	4.5 × 10^8^	+	+	+	+	+	−	−
NR03	Raipur, CG (20.71565 N 81.92652 E)	Rice field Water	2–3 mm, Clear	5.2 × 10^7^	+	+	+	+	+	−	−
NR04	Balod, CG (20.731133 N 81.202309 E)	Rice field Water	2 mm, Clear	6.4 × 10^7^	+	+	+	+	+	−	−
NR05	Raipur, CG (21.2265 N 81.7732 E)	Rice field Soil	2–3 mm, Clear	8.6 × 10^8^	+	+	+	+	+	−	−
NR06	Balaghat, MP (21.812876 N 80.183830 E)	Rice field Water	3–4 mm, Clear	6.4 × 10^7^	+	+	+	+	+	−	−
NR07	Rajnandgaon, CG (20.63934 N 80.73706 E)	Rice field Soil	4–5 mm, Clear	4.8 × 10^8^	+	+	+	+	+	−	−
NR08	Durg, CG (21.190449 N 81.284920 E)	Rice field Water	1–2 mm, Clear	6.2 × 10^9^	+	+	+	+	+	+	−
NR09	Durg, CG (21.229767 N 81.335884 E)	Rice field Soil	1–2 mm, Clear	8.4 × 10^8^	+	+	+	+	+	−	−
NR10	Hyderabad, TS (17.387140 N 78.491684 E)	Rice field Water	8–9 mm, Clear	5.8 × 10^8^	+	+	+	+	+	−	−
NR11	Kawardha, CG (21.909931 N 81.251883 E)	Rice field Water	9–10 mm, Clear	8.2 × 10^7^	+	+	+	+	+	−	−
NR12	Surajpur, CG (23.2378 N 82.7932 E)	Rice field Debris	4–5 mm, Clear	5.8 × 10^8^	+	+	+	+	+	+	−
NR13	Korba, CG (22.6190 N 82.5429 E)	Rice field Soil	7–8 mm, Clear	7.3 × 10^7^	+	+	+	+	+	−	−
NR14	Dantewada, CG (18.900764 N 81.345177 E)	Rice field Water	6–7 mm, Clear	3.4 × 10^6^	+	+	+	+	+	−	−
NR15	Baloda Bazar, CG (21.656917 N 82.159196 E)	Rice field Water	2–3 mm, Clear	4.5 × 10^7^	+	+	+	+	+	+	−
NR16	Raipur, CG (21.4058491 N 81.9261929 E)	Rice field Soil	3–5 mm, Clear	5.3 × 10^9^	+	+	+	+	+	−	−
NR17	Raipur, CG (21.37366 N 81.81014 E)	Rice field Soil	3–4 mm, Clear	4.6 × 10^9^	+	+	+	+	+	−	−
NR18	Bilaspur, CG (22.078642 N 82.152328 E)	Rice field Water	2–3 mm, Clear	7.5 × 10^7^	+	+	+	+	+	+	−
NR19	Sukhma, CG (18.391134 N 81.659273 E)	Rice field Debris	3–5 mm, Clear	3.6 × 10^7^	+	+	+	+	+	−	−

+ indicates lytic to bacteria tested; − indicates not lytic to bacteria tested; PFU, plaque forming count.

### Isolation of *Xanthomonas oryzae* pv*. oryzae*

Diseased rice leaf samples showing typical bacterial leaf blight symptoms were collected from different rice fields during Kharif 2017 from different agroclimatic zones as mentioned earlier in the studied area. Samples were collected based on the random sampling method following standard sampling protocol. The leaf samples were washed with tap water, surface-sterilized with 1% sodium hypochlorite for 30 s, then washed in sterilized distilled water, and air-dried. Surface-sterilized leaf tissues were dried on sterile filter paper and cut into small pieces of approximately 5 mm × 5 mm in size (Bradbury, 1970). Six to seven tissue pieces were placed on peptone sucrose agar (PSA) plates (peptone 10 g; L-glutamic acid 1 g; sucrose 10 g; and agar 15 g per liter) and incubated at 28°C for 2–7 days. Yellow round mucoid colonies convex appearing with smooth margins on the Petri plates were selected and purified three times on PSA as a single colony. Isolates were subjected to Gram staining, potassium hydroxide test (3% KOH; Sulsow et al., 1982), oxidase test, and catalase test and then further confirmed as *Xoo* pathogen by PCR and pathogenicity test as mentioned below. Confirmed *Xoo* pathogens were stored in PSA agar slants at 4°C and 20% glycerol at −80°C for further use.

### Species-specific PCR of *Xoo*

Molecular confirmation of *Xoo* was carried out using the primers *JLXooF* Forward primer (5′-CCTCTATGAGTCGGGAGCTG-3′) and *JLXooR* Reverse primer (5′-ACACCGTGATGCAATGAAGA-3′) encoding *glycosyltransferase* gene. PCR cycling condition includes initial denaturation at 95°C for 5 min, 35 cycles of denaturation at 95°C for 60 s, annealing at 58°C for 45 s, extension at 72°C for 45 s, and final extension at 72°C for 7 min amplifying an internal fragment of 230 bp (Lu et al., 2014).

### Pathogenicity testing of *Xoo* in rice

Rice seeds of the highly susceptible variety TN-1 were sown in a nursery bed, and 3 weeks later, seedlings were transplanted to plastic pots (size 30 cm diameter × 30 cm height). Rice plants were grown under controlled conditions. Bacterial suspensions of *Xoo* isolates were prepared in sterile normal saline at a concentration of 10^8^ CFU/mL and were inoculated by the leaf-clipping method (Kauffman et al., 1973) using sterile scissors in fully expanded leaves of rice plants. Control leaves were treated with sterile normal saline. Lesions on leaves were observed daily for 14 days after inoculation (Backer, 2002). Leaves showing typical symptoms of BLB were processed for re-isolation of the pathogen in PSA plates.

### Bacterial cultures

The *Xoo* pathogen isolated as mentioned earlier namely XooCG-01 was used as a host for the isolation of bacteriophages against *Xoo*. In addition to *Xoo* strains, *Xanthomonas campestris* isolated from cabbage infected with black rot disease and *Bacillus cereus*, *B. subtilis*, *B. thuringiensis*, *Pseudomonas* spp., *and Enterobacter* spp. isolated previously from Chhattisgarh at ICAR-NIBSM were used to determine the host range of phages.

### Isolation and purification of bacteriophages

Bacteriophages against *Xoo* were isolated by an enrichment method (van Twest and Kropinski, 2009) with slight minor modifications from rice field water, soil, and plant debris samples (*n* = 147) collected from the rice fields of Chhattisgarh state (119), and its adjoining seven states Jharkhand (*n* = 7), Uttar Pradesh (*n* = 6), Odisha (*n* = 5), Madhya Pradesh (*n* = 3), Andhra Pradesh (*n* = 2), Maharashtra (*n* = 2), and Telangana state (*n* = 2) during Kharif 2019. Approximately 5 gm of soil or 5 gm of triturated plant tissue debris samples were dissolved in 50 mL of nutrient broth and incubated at 28°C for 1 h and then centrifuged while rice field water samples were centrifuged directly to remove the soil sediments and large coarse particles. In brief, 5 mL of the log phase culture of *Xoo* grown in PS broth (peptone 10 g; sucrose 10 g; sodium glutamate 1 g; and distilled water 1,000 mL) was added to 10 mL of 5X strength PS Broth, and then, 35 mL of the sample supernatant was added and incubated at 28°C for 5 days. On days 2 and 5, a 25 mL aliquot of processed sample was centrifuged to clarify bacterial culture and the supernatant was filtered through a 0.22 μm PVDF filter (Merck Millipore).

For bacteriophage isolation, the filtrate was inoculated in triplicates by agar overlay technique on PSA double-layer plates containing solid (1.5% agar) and semi-solid (0.7% agar) PS medium supplemented with the bacterial host and incubated at 28°C for 5 days until the formation of plaques (van Twest and Kropinski, 2009; Jain et al., 2015). Plates having plaque formation (clear lysis, appreciable by naked eyes, on *Xoo* culture lawn) were preserved, and the plaques were confirmed for the presence of phage by secondary streaking. The phage plaques were picked up with a sterile toothpick and were purified by thrice successive single plaque isolation methods to achieve pure phage isolates. Pure plaques were collected with a sterile toothpick and transferred to *Xoo* culture broth. After incubation at 28°C for 24 h, the bacterial cells were removed through centrifugation and the supernatant was filtered through a 0.22 μm sterile filter.

### Preparation of phage stocks

Phage stocks were prepared from semi-confluent growth on several soft agar plates obtained by inoculating diluted purified phage solutions and incubated for 28°C. After 48 h, plates with a semi-confluent lawn of plaques were added with 3 mL of SM buffer and incubated at 4°C overnight (Shi et al., 2012; Jain et al., 2017). The SM buffer from all plates was collected into a 50 mL polypropylene tube and centrifuged at 7,000 rpm for 10 min to pelletize the bacterial cell. The supernatant was filtered through 0.22 μm sterile filtration units and stored at 4°C till further use.

### Bacteriophage titer determination

To determine the concentration of bacteriophage in the stock filtrate, 100 μL of the filtrate was added to 900 μL of PBS buffer (pH 7.4) and 10-fold serial dilutions were made in PBS buffer. In brief, 100 μL from each dilution was subjected to plaque formation for 24 h using the double-layer agar method. The concentration of each bacteriophage in the stock filtrate was expressed as plaque forming units (PFU/mL).

### Determination of host range of phages

The lytic activity of isolated phages was tested on six different isolates of *Xanthomonas oryzae* pv*. oryzae*, bacterial isolates of *X. campestris*, *Bacillus cereus*, *B. subtilis*, *B. thuringiensis*, *Pseudomonas* spp., and *Enterobacter* spp. isolated at ICAR-NIBSM during 2015–2020. The streak test method was used as a rapid and efficient method for determining the host range. In brief, 200 μL individual bacterial strains in the log phase were added individually to tubes containing 5 mL of 0.7% soft agar. The suspension was transferred to a Petri dish with PSA or NA and allowed to solidify. All the phage isolates were streaked individually as a single line on agar plates, which were then incubated at 28°C for 48 h. The experiment was performed in duplicate.

### Transmission electron microscopy

A drop of 3 μL purified high-titer phage (≥10^8^ PFU/mL) was deposited on a carbon coated grid and allowed to adsorb for 1 min, followed by 2% phosphotungstic acid (PTA; pH 6.8–7.0) and allowed standing for 2 min. The excess PTA was drained off, dried, and observed under a transmission electron microscope (Model: CRYO-TEM TALOS S; Make: Thermo Scientific) under 80 kV at Sophisticated Analytical Instrument Facility (SAIF), All India Institute of Medical Science (AIIMS), New Delhi, India. Images of phages were acquired using Digital FluCam Ceta 16 M Camera. The dimensions of capsid diameter and the tail length of at least six phage particles of each phage sample were measured to calculate the average and standard error values using the software ImageJ (Ver. 1.53e). Based on the TEM morphology, bacteriophages were classified into their respective families as per the guidelines of the International Committee on Taxonomy of Viruses (ICTV; Ackermann, 2011) and named following the phage nomenclature defined by Kropinski et al. (2009).

### Effect of temperature, pH, direct sunlight, UV radiation, and chemicals on the viability of phage NR08

Purified phage NR08 in SM diluent having titer 6.2 × 10^9^ PFU/mL was exposed to different physicochemical conditions (pH, temperature, direct sunlight, and UV-C radiation) and then titrated against host bacteria using the soft agar overlay method. To determine the heat stability, purified phage preparation was exposed to various temperatures, *viz.*, 4°C, 25°C, and 40°C to 90°C on a gradient thermocycler for 15 min and then titrated (Lim et al., 2013). In the pH stability assay for NR08 phage, 100 μL of phage preparation was added to 900 μL SM buffer adjusted with NaOH or HCl to a pH range of 2–12 and incubated at room temperature for 18 h and then titrated (Marquioni et al., 2022). The effect of ultraviolet (UV) irradiation was also studied, for which as a source of UV light, a UV lamp integrated into the laminar flow hood was used (Philips UV light 36 Watt G36T8, UVC = 254 nm). Phage NR08 was diluted in SM buffer to an initial titer of 10^6^ PFU/mL, and 1 mL each was poured into two Petri dishes. Phage samples were exposed to UV irradiation from a distance of 90 cm for the duration of 2 and 5 min, and then, the titer of phages in exposed plates was assayed (Zaczek-Moczydłowska et al., 2020). To determine the direct effect of sunlight on the viability of phage NR08, 1 mL the purified phage preparation (~10^8^ PFU/mL) was poured into a sterile Petri plate with cover and 20 such plates were kept in the open field under direct sunlight at 11:00 a.m. Indian standard time in the month of December 2020. At an interval of every 15 min, one plate was collected and the count of viable phages in exposed plates was assayed. Similarly, the effect of some chemicals, SDS (1%), phenol (5% aqueous), and formalin (40%) on the survivability of phages was also observed (Pandey et al., 2013). The purified phage preparation was mixed with an equal volume of the test chemical and incubated for 1 h at 28°C with intermittent shaking and then subjected to phage titration.

Percentage reduction in phage titer was calculated using the following formula:

Percentage titer reduction = (Phage original titer − Phage titer after treatment) × 100/Phage original titer

### Adsorption rate and one-step growth curve

To determine the infection cycle of phage, the one-step growth curve of phage vB_XooS_NR08 was drawn following the protocol (Owen et al., 2017). One mL of phage NR08 (1 ×10^8^ PFU/mL) was added to the 10 mL of the early-log phase of the XooCG01 culture (1 ×10^8^ CFU/mL) at 0.1 multiplicity of infection (MOI) and incubated at 28°C for 5 min. The unabsorbed phages were then removed through centrifugation at 14,000 rpm for 1 min. The phage titer (unabsorbed phages) in the supernatant was determined using the double layer agar plate method to calculate the absorbance rate. Thereafter, the precipitate was reconstituted into 50 mL of fresh PSB and cultured at 28°C. One mL of sample was withdrawn at the interval of every 10 min and centrifuged at 14,000 rpm for 1 min. The supernatants were processed for phage count at each time point to evaluate the count of free phage using the double-layer agar plate method.

The adsorption rate of phage was calculated as follows:

Adsorption rate = (Initial phage titer − unabsorbed phages titer)/Initial phage titer

A one-step growth curve was plotted, and the latent period, rise period, and phage burst size of phage NR08 were calculated as previously described by Owen et al. (2017). The latency period is the time from the adsorption of the phage on bacterial cells to the release of new phages from the host bacterial cells. The burst period refers to the period from the beginning of phage release after latency to the end of phage release. The burst size was calculated as the ratio of the final count of released phage particles to the initial count of infected bacterial cells at the time of infection.

### Protein profile

The structural proteins of vB_XooS_NR08 phage were separated and observed using sodium dodecyl sulfate polyacrylamide gel electrophoresis (SDS-PAGE). High-titer phages were precipitated overnight at 4°C using 30% polyethylene glycol (PEG-8000) and 3 M NaCl in the ratio of 2:1 and then centrifuged at 14,000 rpm for 15 min. The pellet was reconstituted in SM buffer to concentrate one-tenth of the initial volume of phage and then subjected to SDS-PAGE as per the method of Laemmli (1970) using 10% resolving gel of SDS-PAGE and Tris-glycine as running buffer. The concentrated phage samples were mixed with 2X sample buffer and boiled at 100°C in a water bath for 10 min. A standard broad range protein molecular weight marker (Promega) with a range of 10 to 210 kDa was used to estimate the molecular weights. The gel was stained with Coomassie Brilliant Blue R250 dye and destained with the destaining solution for 30 min, and then, the gel was visualized and documented using the Gel Doc system (Protein simple, Alfa Innotech Corporation, United States).

### Phage nucleic acid extraction

To remove bacterial DNA and RNA contamination prior to phage genome recovery, high-titer NR08 phage suspension (~10^8^ PFU/mL) was treated with DNase I and RNase A for 2 h at 37°C. Thereafter, both the enzymes were inactivated by treating with EDTA at room temperature for 10 min followed by proteinase K treatment for 2 h at 55°C. High-titer phage was precipitated using 30% polyethylene glycol (PEG-8000) and 3 M NaCl as discussed for protein profiling. The PEG-concentrated phage pellet suspended in SM buffer was used for phage nucleic acid extraction using Wizard Genomic DNA Purification Kit (Promega) in accordance with manufacturer protocols. Nucleic acid integrity and absence of degradation were verified by 0.8% agarose gel electrophoresis. Furthermore, nucleic acid was treated with DNase I, RNase A, and S1 nuclease to identify the type of nucleic acid present in the NR08 phage.

### Phage genome sequencing, assembly, and annotation

The quality and quantity of isolated phage DNA were analyzed on a high-sensitivity DNA electrophoresis chip run on Agilent Bioanalyzer 2100 (Agilent Technologies, United States). The genome sequencing library was prepared for pair end sequencing using Illumina HiSeq following the manufacturer protocol, and qualitative and quantitative analyses of the library were performed using an Agilent Bioanalyzer 2100 (Agilent Technologies, United States). The phage’s genomic DNA libraries were sequenced using the Illumina sequencing approach on Illumina HiSeq 2500 machine at Nucleome Informatics Pvt. Ltd., Hyderabad. The raw reads generated by sequencing of phage genomic libraries were cleaned and quality-trimmed using the FASTP software (Chen et al., 2018). The high-quality filtered reads of phage were used for the *de novo* assembly using the SPAdes assembler v3.14 (Bankevich et al., 2012) using the “careful mode” which includes reading correction module, reduces the mismatches and false indels arising during the assembly and k-mer-titration based on read characteristics. The *de novo* assembly was also performed using SKESA assembler v2.3.0 (Souvorov et al., 2018) with default parameters. The assemblies are finally chosen on the basis of the following parameters: (i) N50; (ii) number of contigs; (iii) maximum contigs size; and (iv) number of bases in the assembly. The assembly generated by SPAdes produced more N content and gaps, while the assembly produced by SKESA-assembler did not contain N and gap. Therefore, SKESA-assembled genome assembly was further used for annotation and analysis.

The assembled contigs were used for the functional annotation using RASTtk (Overbeek et al., 2014; Brettin et al., 2015), and tRNA detection was performed using the software tRNAscan-SE v. 2.0 (Schattner et al., 2005) and ARAGORN (Laslett and Canback, 2004) using with default settings. Functional annotation was also performed using Blast2GO 6.0.3 (Conesa et al., 2005). The predicted genes were used to search the NCBI non-redundant database, the conserved domain database, the Cluster of Orthologous Groups database, and the InterPro database and were annotated using Blast2GO V.0.3. Automated annotation performed by RASTtk was manually curated by individually analyzing each predicted gene using Blast2GO. Phage life style predication was performed using the online tool phage AI^1^ to predict phage lifestyles.

### Comparative genome analysis

The assembled genome of Phage NR08 was compared with the NCBI databases using BLASTn (Altschul et al., 1990). The blast was done using BLASTN 2.12.0^+^ (Zheng et al., 2000) with NCBI databases, and the results were visualized using the Kablammo: an interactive, web-based BLAST results using http://kablammo.wasmuthlab.org. Comparative genome analysis was conducted by whole-genome alignment with 12 previously reported Xoophages having genome sequences available with NCBI. Genome sequences of all the previously reported Xoophages were downloaded which includes 10 Xoophages namely XPP/XPV (Accession nos. MG944227 to MG 944236; Kovács et al., 2019), OP2 DNA (Acc. no. AP008986; Inoue et al., 2006), and Xoo-sp2 (Acc. No. KX241618; Dong et al., 2018) and aligned using CLC Genomics Workbench V 20.0.4 with the default parameters and visualization of comparative map and genome map of NR08 phage. Average nucleotide identity comparison 1.0 was performed with a default setting minimum similarity fraction of 0.8 and minimum length fraction of 0.8.

### Phylogenetic analysis

To define the evolutionary history of the new phage species, a phylogenetic analysis was performed. Nucleotide sequences of NR08 phage were compared with the NCBI database using BLASTn. The whole-genome data of phages having the highest homology with NR08 were downloaded from NCBI, and a phylogenetic tree was constructed using the neighbor-joining method using Jukes-Cantor nucleotide distance measure with 500 bootstrap replicates using CLC Genomics Workbench v. 20.0.4.

### Bacterial challenge assay

One mL of XooCG01 (10^8^ CFU/mL) at exponential growth phase was co-inoculated with 1 mL of NR08 phage (10^7^ PFU/mL) at 0.1 MOI in a culture flask into 50 mL of PSB, while another flask, with the same volume of PSB, inoculated with 1 mL of XooCG01 (10^8^ CFU/mL) only, was kept as bacterial control. Both the flasks were incubated at 28°C for 48 h. At every 6-h interval, 500 μL of the medium was taken out from both the flask and processed for total colony count of host bacteria by 10-fold serial dilution method and plated in PSA plates in triplicate and incubated at 28°C for 72 h. The total bacterial colony count of every collect was evaluated in terms of CFU/mL for both bacterial control and phage-treated. The final bacterial concentrations were compared between phage inoculated and bacterial control cultures to determine the effect of phage in reducing bacterial growth. The percent decrease in total bacterial count in phage-treated was calculated as compared to the bacterial control.

### Protective efficacy of vB_XooS_NR08 phage in rice pot

To determine the protective efficacy of vB_XooS_NR08 phage against bacterial leaf blight infection, rice seedlings were transplanted in pots (size 30 cm diameter × 30 cm height) having five plants in each pot and each treatment was replicated three times. The experiment was arranged in a completely randomized block design at environmental temperature. At the maximum tillering stage, rice plants were infected with *Xanthomonas oryzae* pv*. oryzae* pathogen at a concentration of around 10^8^ CFU/mL using the leaf clip inoculation method in highly susceptible TN-1 variety as previously described (Kauffman et al., 1973). Briefly, the 3-day-old grown XooCG01 culture on PSA was harvested in sterile saline solution, and suspension turbidity was adjusted to obtain a concentration of 10^8^ CFU/mL to infect the rice plants by clipping five leaves per plant. After 72 h of BLB infection, plants were treated with a single application of selected phage in two preparations: (i) neat NR08 phages (without any supplement or additive) at concentration 10^7^ PFU/mL and (ii) NR08 phage at concentration 10^7^ PFU/mL supplemented with 2% skim milk preparation, using the spray method, whereas the phage untreated plants were sprayed with sterile water and considered as untreated infected control plants. Application of phage preparation was performed with a handheld sprayer during the evening hours of the day. The plants were observed and recorded daily for up to 21 days for the progression of disease symptoms in both the phage-treated and the untreated control groups. The length of lesions on 10 leaves per treatment was recorded and evaluated for each group and used for analysis. The disease suppression efficacy of the phage-treated group was compared with the untreated control group.

### Statistical analysis

The data were subjected to analysis of variance (ANOVA) using the online Indian NARS statistical computing portal at the IASRI server.^2^ Statistical significance was evaluated using Duncan’s multiple range test (DMRT), and the *p*-value <0.01 was considered significantly different.

## Results

### Isolation and characterization of *Xoo*

**Figure 1:**
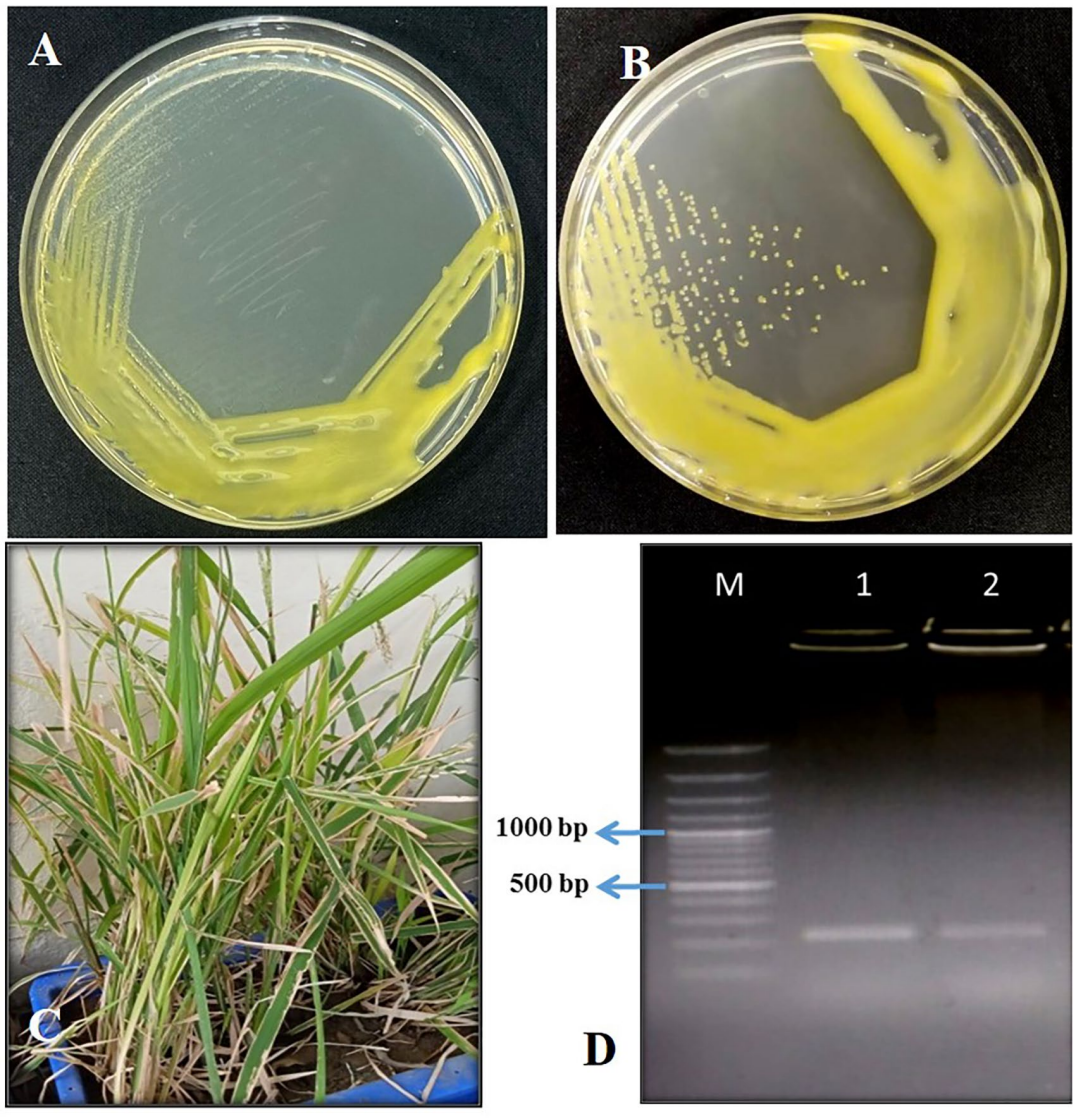
**(A)** Growth of *Xoo* culture straw yellow color smooth entire margin after 3 days; **(B)** slimy and glistening colony after 5-day incubation; **(C)** pathogenicity testing of *Xoo* isolates in rice pots with symptoms of BLB infection; **(D)** confirmation of *Xoo* by JLXoo-1 primer PCR with captions M: 100 bp Ladder, 1: *Xoo* culture, 2: *Xoo* re-isolated after pathogenicity test in the rice pot.

The leaves showing typical symptoms of bacterial blight were collected from rice fields in five states of India. Twenty-four bacterial isolates were obtained from the infected leaf samples on PSA plates with typical characteristics of *Xoo*, i.e., yellow, round, mucoid colonies convex appearing with a smooth margin. Isolates with Gram-negative, KOH positive, oxidase negative, and catalase positive reactions were further confirmed as *Xoo* pathogens by PCR and pathogenicity test. All 24 isolates were further subjected to *Xoo*-specific PCR and pathogenicity test. Six isolates were confirmed as *Xanthomonas oryzae* pv*. oryzae* which produced 230 bp PCR product using *Xoo*-specific PCR primers and showed typical symptoms of BLB in pathogenicity test in rice. In the pathogenicity test, on 5 days post-inoculation, symptoms started appearing with the formation of water soaked lesions from the margin, and, by 10 dpi, lesions were produced all along the leaf blade in a wavy pattern extending in length and width (Figure 1). These six isolates belonging to states Chhattisgarh (3), Odisha (2), and Tripura (1) were named XooCG01, XooCG02, XooCG03, XooOD01, XooOD02, and XooTR01. Among these six isolates, XooCG01 showed maximum pathogenicity in terms of the production of leaf lesions, disease severity, and wilting of plants. Therefore, the XooCG01 strain was used as the host strain for phage isolation and further experiments.

### Isolation and characterization of phages

**Figure 2:**
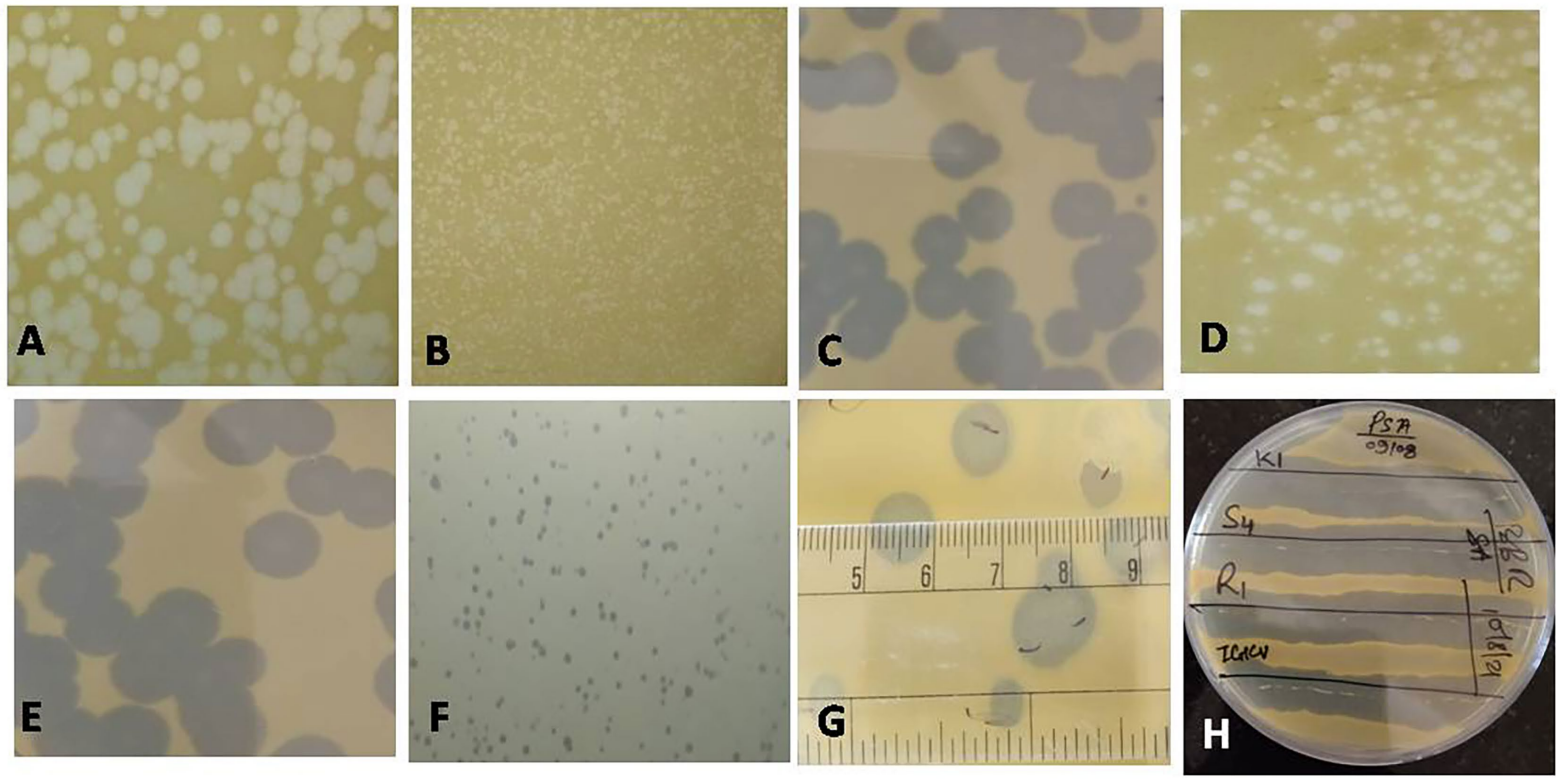
Plaques of different phages measuring between 1 and 10 mm in size (**A**: NR16; **B**: NR08; **C**: NR13; **D**: NR02; **E**: NR10; **F**: NR09; **G**: NR11); **(H)** Clearance of growth around phage streaked lines on *Xoo*-inoculated soft agar plates for host range.

Water, soil, and plant debris present in rice fields provide an enriched composition of the bacterial population, making it an excellent environment for the isolation of bacteriophages. Out of 147 samples collected, we isolated and purified 19 bacteriophages of *Xanthomonas oryzae* pv*. oryzae* from rice field water (11), soil (6), and field debris (2) using phage enrichment protocol representing three states of India, *viz.*, Chhattisgarh (17), Madhya Pradesh (1), and Telangana state (1). These isolated Xoophages were designated as NR01 to NR19 (Table 1). The maximum recovery of phages was from samples belonging to *Xoo*-infected field, while only one phage NR14 was isolated from uninfected field water. These phages produced clear round lytic plaques of size ranging from 1 to 10 mm in diameter (Figure 2). Based on the plaque size, phages were grouped into three categories, *viz.*, small size plaque producers (1–3 mm), medium size plaque producers (3–6 mm), and large size plaque producers (6–10 mm) containing eight, seven, and four phages, respectively. Among all phages, the smallest size plaques were observed in phages NR08 and NR09, while the largest plaques were formed by phage NR11. The titer of the isolated phages varied from 3.4 × 10^6^ to 6.2 × 10^9^ PFU/mL with the lowest and highest in NR14 and NR08 phages, respectively (Table 1).

### Host range

All phages were able to lyse *Xoo* strains and displayed the same host range, showing the lytic activity for tested five *Xoo* isolates namely XooCG01, XooCG02, XooCG03, XooOD01, and XooOD02 except XooTR01, forming clear zone around the phage streaked lines on the bacterial lawn of each tested strain, respectively, while none of the phages showed any lytic activity for *X. campestris* and other heterologous host bacterial species used in this study, *viz.*, *Bacillus cereus*, *B. subtilis*, *B. thuringiensis*, *Pseudomonas* spp., and *Enterobacter* spp. (Table 1) suggesting the isolated phages were highly specific to their bacterial host *Xoo*.

The host specificity of phages revealed that all the 19 isolated Xoophages infected all five *Xoo* strains isolated from the state of Chhattisgarh and Odisha, while only four phages (phages NR08, NR12, NR15, and NR18) possess infectivity for *Xoo* strain belonging to state Tripura. This might be due to variation in the binding capability of phages to host XooTR01 strain isolated from the northeastern state of India, whereas none of the phages were able to infect the bacteria other than *Xoo* indicating their high host specificity and, thus, will not harm the beneficial microflora present in the soil when further used as a biocontrol agent against *Xoo*. Phage NR08 has a broad range of *Xanthomonas* host strains, as it infected all the six strains of *Xoo* tested and was also found to have the highest titer, hence, the phage NR08 was used for further studies.

### Phage morphology *via* TEM

**Figure 3:**
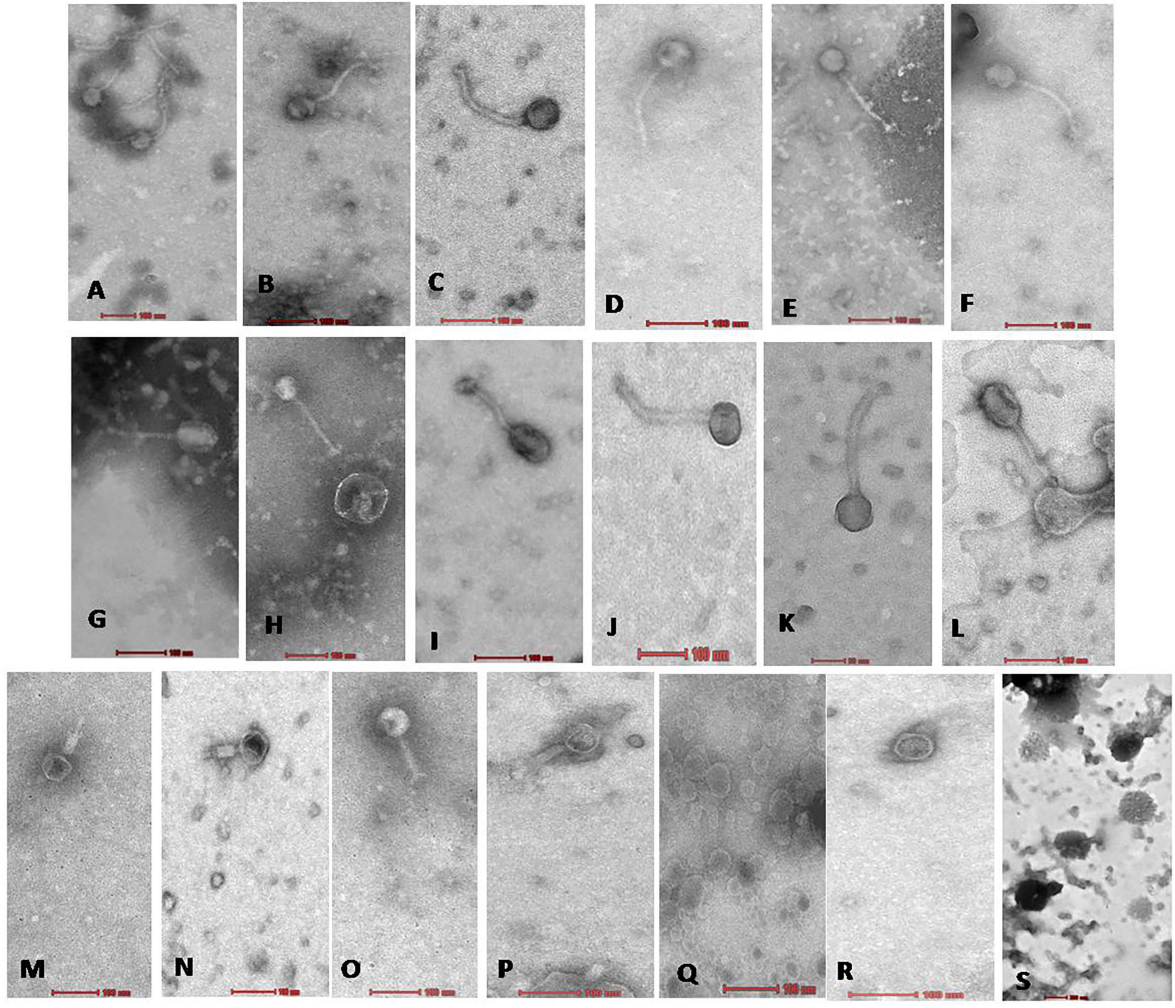
Electron micrograph of all 19 Xoophages with head and tail morphology belonging to families *Siphoviridae* **(A–L)**; *Myoviridae* **(M–P)**; *Podoviridae* **(Q,R)**; and Unclassified **(S)**. From **(A−S)**, all 19 phages are as follows **A**: NR02; **B**: NR04; **C**: NR07; **D**: NR08; **E**: NR09; **F**: NR10; **G**: NR11; **H**: NR12; **I**: NR13; **J**: NR16; **K**: NR17; **L**: NR18; **M**: NR01; **N**: NR03; **O**: NR06; **P**: NR15; **Q**: NR05; **R**: NR19; **S**: NR14.

On transmission electron microscopy, all 19 Xoophage isolates revealed head and tail morphology belonging to the order *Caudovirales* with representatives of different morphotypes families, *viz.*, *Myoviridae* (4), *Siphoviridae* (12) *Podoviridae* (2), and unclassified (1) indicating that a substantial diversity of phages was isolated during this study. Among the four myoviruses, having a contractile tail, the head width ranged from ~52 to ~64 nm, head length ranged from ~55 to ~68 nm, and tail lengths varied between ~96 and ~190 nm with A1 morphotype. In few electron micrographs of phages, the contractile stage of the phage tail was also observed. Analysis of 12 siphoviruses, having a non-contractile tail, revealed head width ranged from ~51 to ~98 nm, head length ranged from ~58 to ~122 nm, and tail length ranged from 132 to ~212 nm. Out of 12 siphoviruses, morphotypes B1 and B2 were observed in six phages each. While, in the two podoviruses, having a non-contractile short stubby tail, the head width ranged from ~32 to ~44 nm, head length ranged from ~36 to ~45 nm, and tail length ranged from ~12 to ~15 nm with C1 morphotype (Figure 3 and Table 2). In some of the siphoviruses and the myoviruses phages, tail tubes, tail plates, and tail filaments were also visible. Based on the TEM morphology, bacteriophages were classified into their respective families as per the guidelines of the International Committee on Taxonomy of Viruses (ICTV; Ackermann, 2011) and named following the phage nomenclature defined by Kropinski et al. (2009).

**Table 2:** Morphology details and nomenclature of phage on the basis of transmission electron microscopy.

Phage code	Head width (nm) Mean ± SD	Head length (nm) Mean ± SD	Tail length (nm) Mean ± SD	Phage family as per TEM	Phage morpho-type	Phage nomenclature
NR01	56.42 ± 3.16	59.73 ± 2.04	189.74 ± 8.65	*Myoviridae*	A1	vB_XooM_NR01
NR02	62.86 ± 3.24	70.68 ± 3.52	212.58 ± 17.53	*Siphoviridae*	B1	vB_XooS_NR02
NR03	64.37 ± 2.58	67.33 ± 2.37	96.42 ± 6.72	*Myoviridae*	A1	vB_XooM_NR03
NR04	53.72 ± 2.43	64.74 ± 3.65	179.48 ± 12.69	*Siphoviridae*	B2	vB_XooS_NR04
NR05	43.36 ± 3.85	45.85 ± 3.94	14.72 ± 3.42	*Podoviridae*	C1	vB_XooP_NR05
NR06	52.63 ± 2.18	55.28 ± 3.43	115.83 ± 7.92	*Myoviridae*	A1	vB_XooM_NR06
NR07	62.82 ± 2.64	65.36 ± 4.82	172.78 ± 10.87	*Siphoviridae*	B1	vB_XooS_NR07
NR08	56.84 ± 2.58	58.72 ± 2.16	156.42 ± 8.63	*Siphoviridae*	B1	vB_XooS_NR08
NR09	56.34 ± 3.32	64.92 ± 4.38	159.71 ± 9.58	*Siphoviridae*	B1	vB_XooS_NR09
NR10	97.28 ± 5.18	121.89 ± 8.74	132.46 ± 7.92	*Siphoviridae*	B2	vB_XooS_NR10
NR11	51.68 ± 4.26	85.69 ± 6.32	134.93 ± 12.37	*Siphoviridae*	B2	vB_XooS_NR11
NR12	54.74 ± 3.84	62.83 ± 3.73	168.65 ± 15.48	*Siphoviridae*	B1	vB_XooS_NR12
NR13	52.56 ± 2.14	68.46 ± 5.39	198.83 ± 17.66	*Siphoviridae*	B2	vB_XooS_NR13
NR14	286.89 ± 12.65	314.52 ± 18.78	78.35 ± 5.46	Unclassified	*----*	vB_XooUn_NR14
NR15	58.26 ± 3.42	59.37 ± 4.86	137.43 ± 3.27	*Myoviridae*	A1	vB_XooM_NR15
NR16	61.92 ± 1.54	63.62 ± 4.05	206.37 ± 22.46	*Siphoviridae*	B1	vB_XooS_NR16
NR17	56.29 ± 2.03	68.43 ± 3.58	195.87 ± 9.53	*Siphoviridae*	B2	vB_XooS_NR17
NR18	59.82 ± 4.25	72.53 ± 6.76	194.56 ± 9.84	*Siphoviridae*	B2	vB_XooS_NR18
NR19	32.68 ± 3.42	36.92 ± 3.28	12.58 ± 2.86	*Podoviridae*	C1	vB_XooP_NR19

### NR08 phage viability at different environmental conditions

**Figure 4:**
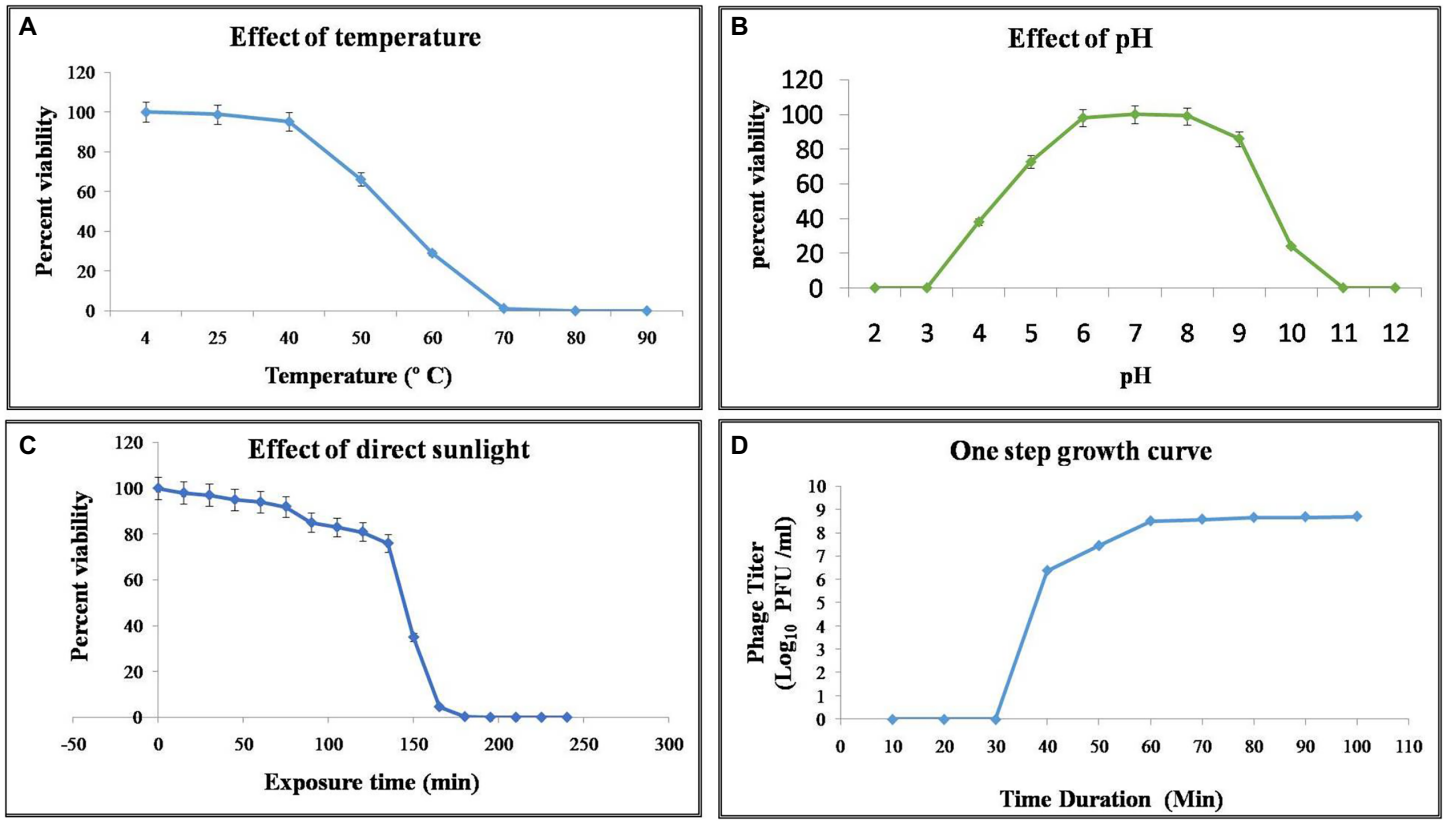
Effect of environmental condition on the viability of phage NR08. **(A)** Effect of temperature on viability; **(B)** effect of pH on viability; **(C)** effect of direct sunlight on viability; and **(D)** one-step growth curve of phage NR08.

Phage stability was assessed for various environmental and chemical factors. NR08 phage was 100% viable at the temperature range of 4°C–40°C, up to 30% viable at 60°C, while less than 1% viability was seen above 70°C indicating the loss of phage viability at elevated temperatures (Figure 4A). Similarly, when tested for pH stability of phage NR08, more than 80% of phages were viable at pH 5–9 as indicated by the remaining phage titer after exposure to this pH range. The phage titer was drastically reduced when incubated at pH below 5 or above 9 as indicated by no detectable effect on bacteriophage survival and infectivity (Figure 4B).

Phage NR08 when tested for UV radiation sensitivity, on exposure duration of 2 and 5 min, the reduction of active phages was up to 6.2 ×105 PFU/mL and almost 100%, respectively. Phage NR08, when exposed to direct sunlight, more than 75% of phages can survive up to an exposure time of 135 min, and thereafter, the viability drastically reduces to less than 1% in exposure duration of 180 min (Figure 4C). Furthermore, all phages were highly sensitive to 40% formalin and 1% SDS showing less than 1% viability, while less than 10% phages were viable after exposure to 5% aqueous phenol.

### Adsorption rate and one-step growth cycle

To enumerate the adsorption rate, phage latent period, burst period, and burst size, we determined a one-step growth curve. Approximately 93% of the NR08 phage particles were adsorbed on the XooCG01 cells in 5 min at 0.1 MOI. The one-step growth curve showed that the phage NR08 had a latent period of 40 min followed by a burst period between 40 and 70 min (Figure 4D). At the end of the burst period, the burst size was approximately 250 virions per infected cell.

### Protein profiling

**Figure 5:**
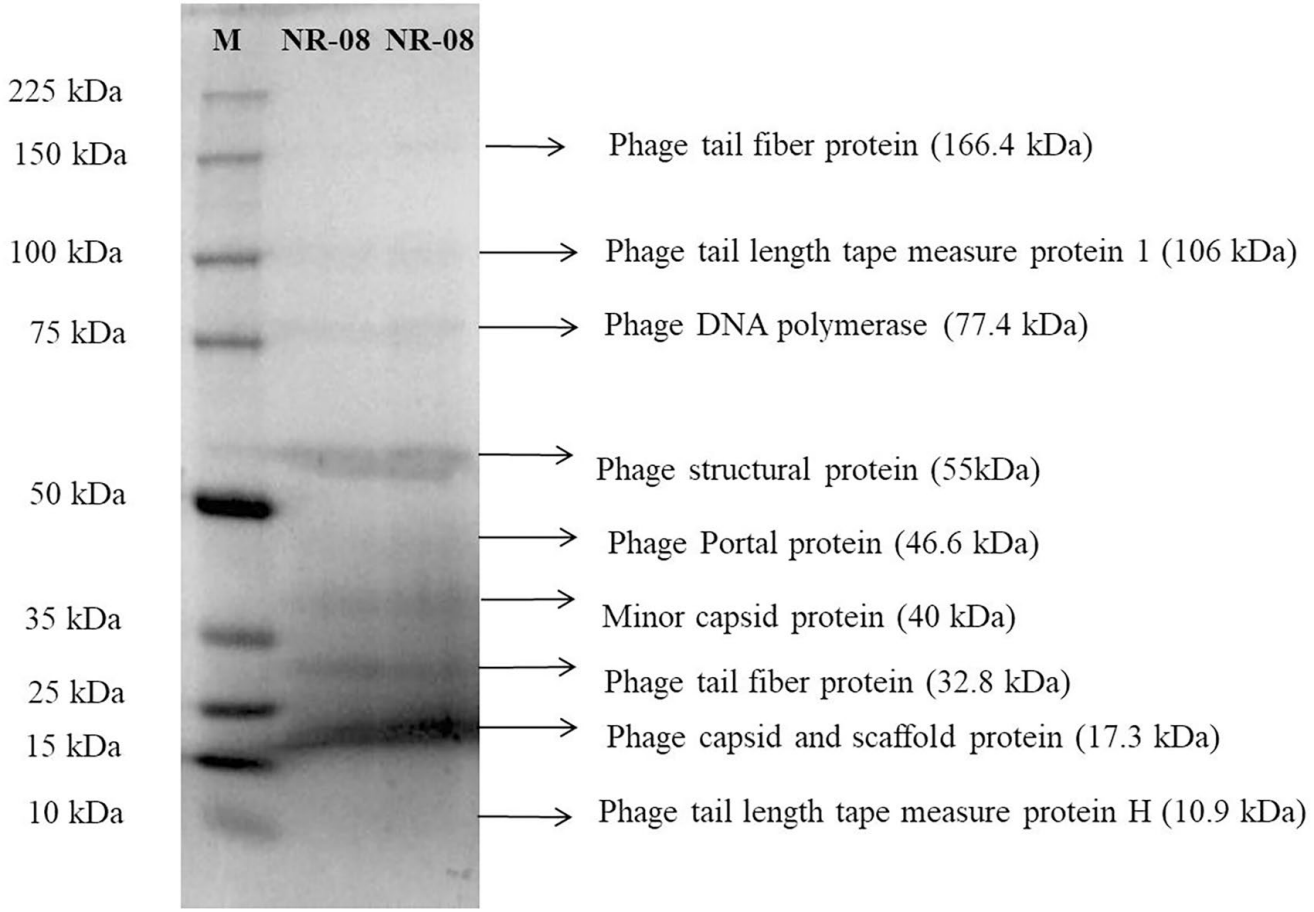
Protein profiling of phage NR08 using SDS-PAGE run along with broad range protein molecular weight markers M (Promega). The SDS-PAGE of phage NR08 showed nine bands of structural protein ranging from 10 to 166 kDa.

Purified phage vB_XooS_NR08 particles were denatured and separated using SDS-PAGE. At least nine distinct protein bands of approximate size 10.9, 17.3, 32.8, 40, 46.6, 55, 77.4, 106, and 166.4 kDa were observed in the Coomassie-stained SDS-PAGE gel, which were expected to be structural proteins of vB_XooS_NR08 by their estimated molecular weights. The predicted proteins may be phage tail fiber proteins, phage tail length tape measure protein 1, phage tail length tape measure protein H, phage DNA polymerase, phage structural protein, phage portal protein, minor capsid protein, phage capsid, and scaffold protein (Figure 5).

### NR08 genomic and proteomic analysis

The nucleic acid of phage NR08 was extracted, and isolated nucleic acid was treated with DNase I, RNase A, and S1 nuclease to identify the nature of nucleic acid which revealed it to be double-stranded DNA. The Illumina sequencing of bacteriophage vB_XooS_NR08 genome generated a total of 16,539,460 pair end reads with a total base of 2662.85 Mb and with an average read length of approximately 161 bp. After quality control and filtering, the total reads remain 12,264,972 with a total base of 1689.24 Mb and average read length of 137.7 bp having Q30 bases of 95.58 and GC content of approximately 52.51%. The detail of sequencing read statistics of raw reads and filtered reads of the phage genome is mentioned in Table 3. The filtered reads were taken for *de novo* assembly using the SKESA and SPAdes genome assemblers. The quality check of contigs showed that the assemblies generated by SPAdes had large gaps and N string, thus, it was not taken for further analysis. The SKESA genome assembler produced a total of 39 contigs with a size of 98,812 bp and a G + C content of 52.9%. The detail of the *de novo* assembly of whole-genome sequences of NR08 using the SKESA assembler is given in Table 3. The complete genome sequence of bacteriophage vB_XooS_NR08 was deposited in GenBank under accession number OQ055246, and the raw sequence reads were deposited in the NCBI database under the BioProject ID: PRJNA844261, BioSample number: SAMN29042521, and SRA accession number: SRR19757545.

**Table 3:** NGS sequencing read statistics of raw reads and filtered reads and *de novo* assembly of whole-genome sequences of NR08 using SKESA assembler.

Phage NR08 sample NGS statistics	Raw reads (before filtering)	High-quality filtered reads (after filtering)
Total reads	16,539,460	12,264,972
Total bases	2,662,853,060	1,689,238,212
Mean read length R1	161	137.7
Mean read length R2	161	137.7
Raw data (GB)	2.66285	1.68923
% GC content	-	52.51
***De novo* assembly of whole genome sequences of NR08 using Skesa**
1.	Total sequence length (bp)	98,812
2.	Number of contigs	39
3.	Average size of contigs	2533.64
4.	GC content (%)	52.9
5.	N50 (bp)	11,437(3)
6.	Longest contig size (bp)	32,457
7.	Gap Ratio (%)	0.000000
8.	Number of genes predicted	142
9.	Number of proteins predicted	142
10.	Number of rRNA	0
11.	Number of tRNA	1
12.	Number of CRISPRS	0
13.	N count	0
14.	Coding ratio (%)	80.4

**Figure 6:**
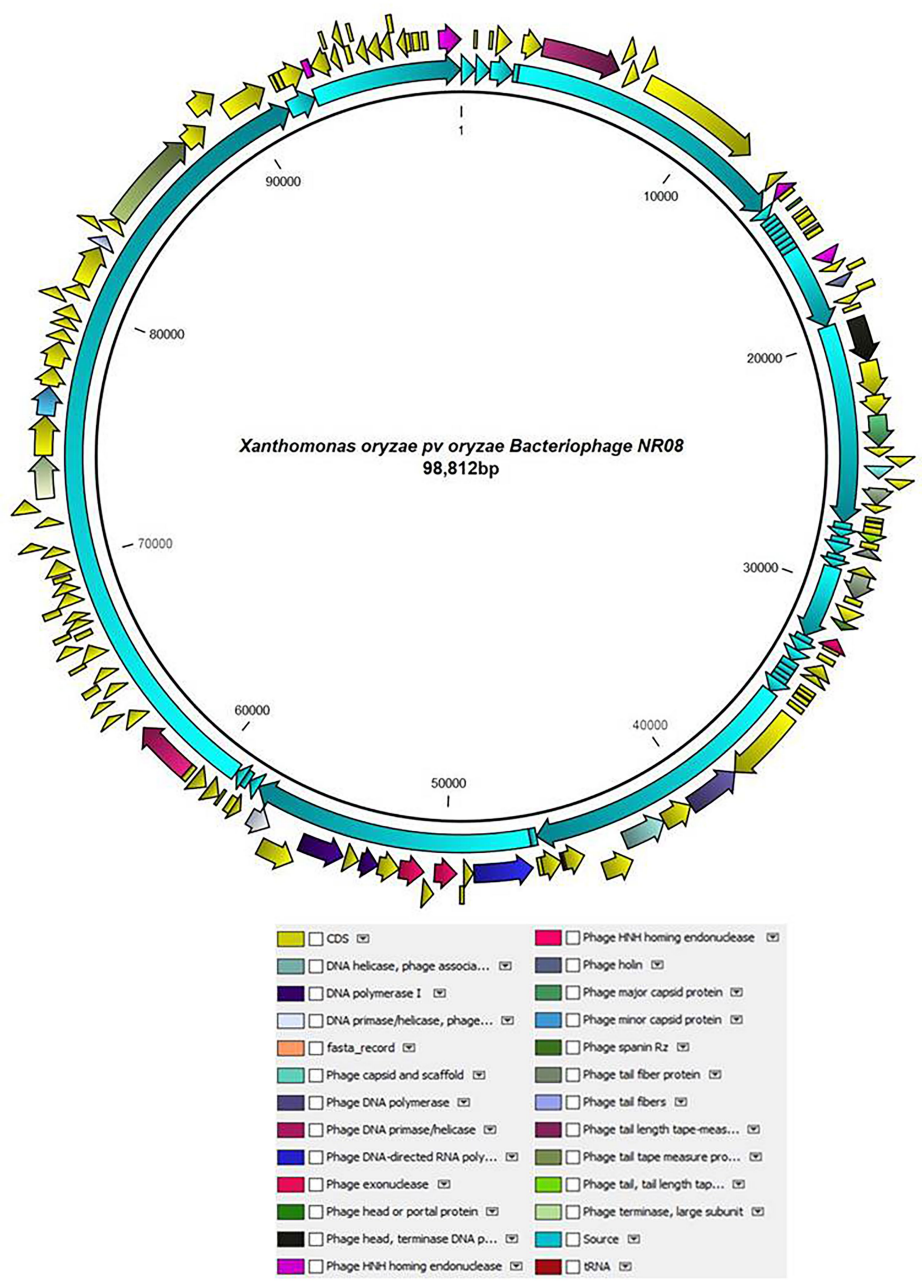
Schematic diagram representing the genome of NR08 phage with the functionally annotated ORF having four different modules of proteins: DNA packaging module, morphogenesis module, DNA replication module, and lysis module.

The whole-genome sequencing analysis of phage NR08 showed the double-stranded linear DNA of 98,812 bp in size with a G + C content of approximately 52.9%. The NR08 genome contains 142 open reading frames (ORFs; Figure 6) and is larger in size than those of previously reported *Xanthomonas* phages (Ogunyemi et al., 2019; Liu et al., 2021; Nazir et al., 2021). The genome assembly was screened for the presence of tRNA using the software tRNAscan-SE v. 2.0 which showed the presence of only one tRNA namely trna1-GlnTTG of 72 bp. Out of 142 ORFs, 83 were present on the positive sense strand while 59 were found on the negative sense strand of the phage genome. Phage lifestyle prediction was done using the phageAI online tool which showed the nature of phage to be a virulent having lytic lifestyle.

### Functional annotation

The functional annotation of the genome sequence revealed that the NR08 phage has 141 probable protein-encoding genes and one gene for tRNA^Gln^ as depicted in Figure 6. Out of 141 protein coding ORFs, 23 ORFs were predicted to code genes for phage structural and host interaction proteins which include virion proteins, major capsid proteins, major tail tube proteins, minor tail proteins, head–tail joining proteins, tail fiber proteins, and tape measure proteins, whereas 42 ORFs have been annotated as hypothetical proteins. Furthermore, bioinformatics analysis revealed that 76 ORFs code for functional proteins of which 32 ORFs were associated with phage metabolism and were involved in DNA replication, recombination, repair, transcription, translation, and other related nucleotide and protein metabolism. Similarly, eight ORFs were related to host lysis function, 12 ORFs were associated with phage assembly and packaging function, and eight ORFs encoding phage portal proteins, chaperones, and scaffold proteins were involved in phage morphogenesis. While 12 ORFs were having endonuclease activity of which seven were having HNH endonuclease function, whereas four ORFs codes for the domain of the unknown function (DUFs domain containing proteins). Out of eight genes associated with host lysis, three ORFs are for holin proteins, two for phage spanin Rz, and one each ORF codes for lysozyme, Clp protease, and peptidoglycan endopeptidase. The complete genome was categorized into four modules based on the functions of encoded proteins in the virion, *viz.*, DNA packaging module, morphogenesis module, DNA replication module, and lysis modules (Figure 6; Supplementary Table 1). Among the 141 ORFs with a predicted structure or function, none were associated with phage lysogenic cycle, antibiotic resistance, toxicity, and allergens.

### Comparative genome analysis

**Figure 7:**
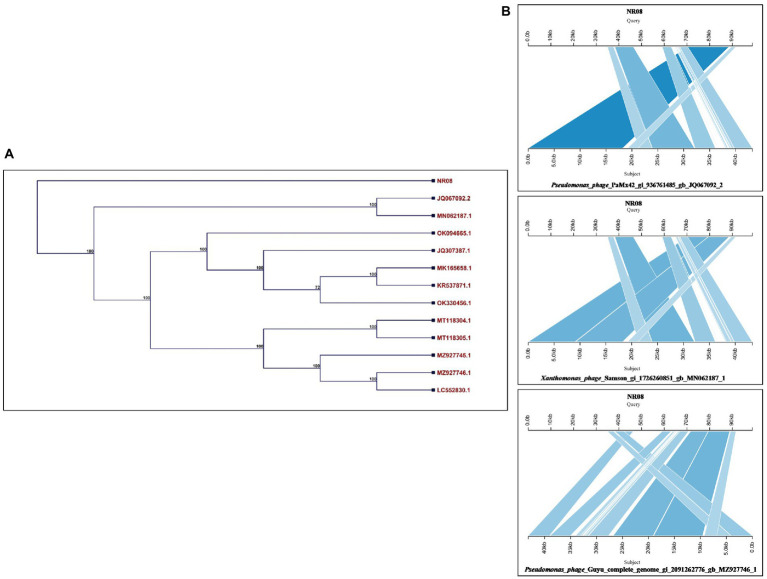
**(A)** Phylogenetic tree based on the whole-genome sequence of vB_XooS_NR08 and its most closely related phages data available with NCBI based on nucleotide identity. A phylogenetic tree was generated using the neighbor-joining method with 500 bootstrap replicates The details of WGS of Xoophages shown in the phylogenetic tree are NR08—*Xanthomonas oryzae* phage vB_XooS_NR08; JQ067092.2—*Pseudomonas* phage PaMx42; MN062187.1—*Xanthomonas* phage Samson; OK094665.1—*Pseudomonas* phage vB_Pae_W3 isolate sewage; JQ307387.1—*Pseudomonas* phage vB_Pae-Kakheti25; MK165658.1—*Pseudomonas* phage vB_PaeS_SCUT-S4; KR537871.1—*Stenotrophomonas* phage vB_SmaS-DLP_2; OK330456.1—*Pseudomonas* phage TehO; MT118304.1—*Pseudomonas* phage Epa40; MT118305.1—*Pseudomonas* phage Epa4; MZ927745.1—*Pseudomonas* phage Kaya; MZ927746.1—*Pseudomonas* phage Guyu; LC552830.1—*Pseudomonas* phage vB_PaeS-Yazdi-M DNA. **(B)** Pairwise BLASTn comparison of the complete genome DNA sequence of NR08 phage with most closely related phages. The gray areas between the genome maps indicate the level of identity at different regions of the genome.

The whole-genome sequence of vB_XooS_NR08 was analyzed using BLASTN analysis against the NCBI non-redundant DNA database. A nucleotide comparison with available genome sequences in the NCBI database showed that out of the top 13 hits, the NR08 phage showed the highest genome similarity with *Pseudomonas phage* PaMx42 Acc. No. JQ067092 (40% query coverage, 95.39% identity, and acc. Length 43,225 bp), *Xanthomonas* phage Samson, Acc. No. MN062187 (40% query coverage, 96.68% identity, and acc. Length 43,314 bp), and *Pseudomonas* phage Guyu, Acc. No. MZ927746 (40% query coverage, 93.74% identity, and acc. Length 43,141 bp). To explore further the evolutionary position of the NR08 phage, a phylogenetic analysis was performed with MEGA using the neighbor-joining method for phage NR08 and its related 12 different phages. The phylogenetic tree showed two major branches based on the whole-genome analysis of these phages indicating NR08 is closely related to three phages, *viz.*, *Pseudomonas* phage PaMx42, *Xanthomonas* phage Samson, and *Pseudomonas* phage Guyu (Figures 7A,B).

The comparative genomic analysis of NR08 phage with previously reported 12 Xoophages was conducted which includes 10 Xoophages namely XPP/XPV (Accession no. MG944227 to MG 944236; Kovács et al., 2019), OP2 DNA (Acc. No. AP008986; Inoue et al., 2006), and Xoo-sp2 (Acc. No. KX241618; Dong et al., 2018). The genome size of these reported Xoophages ranged from 45 to 47 kb except phage Xoo-sp2 which has a genome size of 60.3 kb in length, while the NR08 phage isolated in this study had a genome size of 98.8 kb.

**Figure 8:**
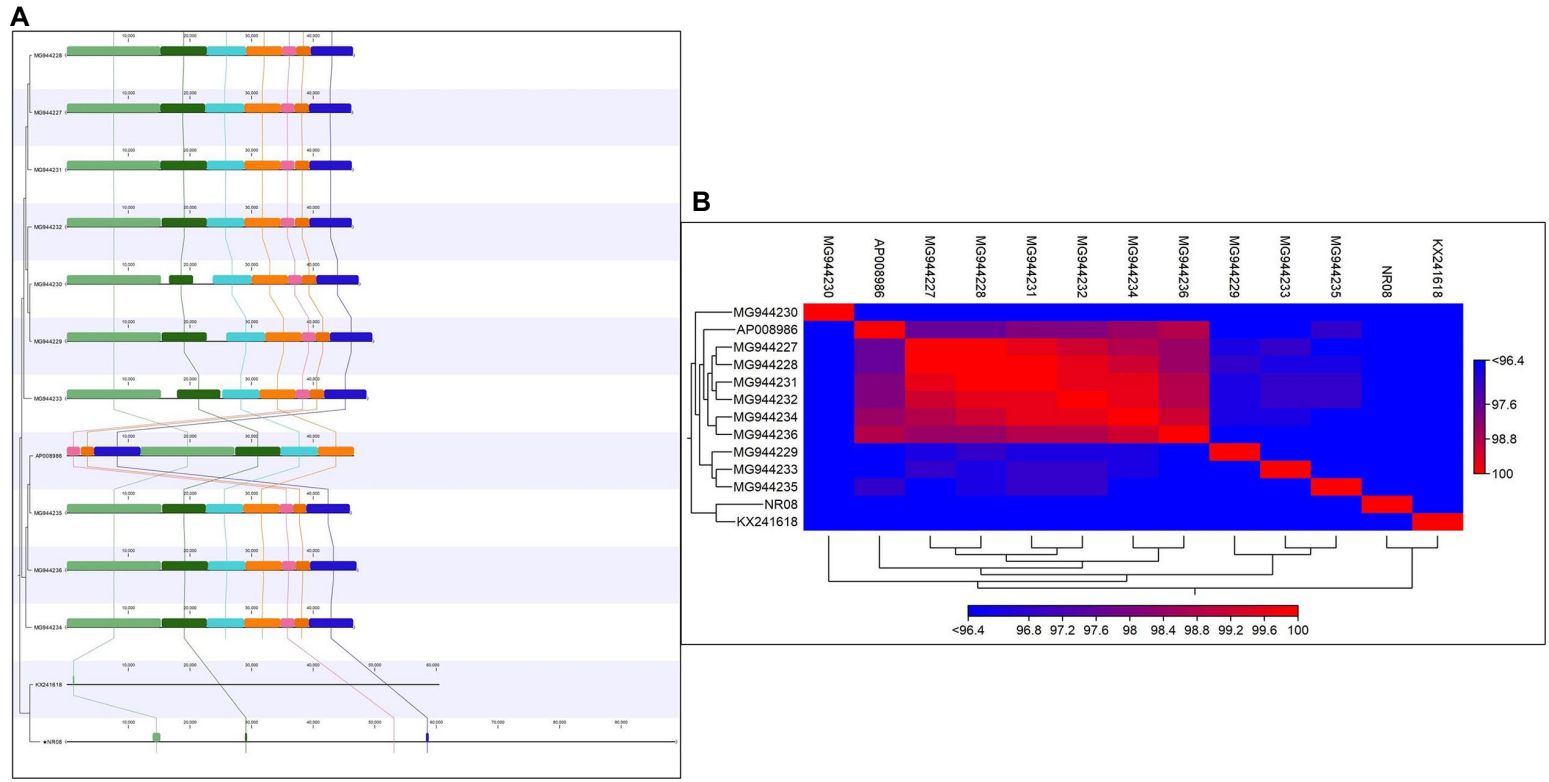
**(A)** Comparative genome analysis of NR08 phage with previously reported 12 *Xanthomonas oryzae* (*Xoo*) phages. The multicolor domains that appeared in the figure depict the homology in different regions within the whole genome of different phages and their physical positions in the genome. **(B)** Heat map showing the phylogenetic relationship of NR08 phage with previously reported *Xanthomonas oryzae* pv *oryzae (Xoo)* phages based on nucleotide sequences.

Average nucleotide identity (ANI) was calculated and found that the ANI was 83.95 with (MG994227-29, 31-33) and 83.44 with AP008986, least or no ANI with (MG994230, 34-36, and KX241618). The average alignment percentage (AP) of NR08 with other Xoophages ranged from 1.19 to 1.25 for MG944227-29, MG944231-33, and AP008986 and 0.70 to 0.71 for MG944230 and MG944234-36 and least AP 0.32 for KX241618 phage (Figures 8A,B). This shows that the most of genome sequence information is novel for bacteriophage, vB_XooS_NR08, infecting *Xanthomonas oryzae* pv*. oryzae*. The genome comparison showed that the matched genome sizes were approximately 43–44 kb out of the 98.8 kb genome size of NR08.

### KEGG mapping of phage genome

KEGG mapping of the phage genome revealed that it codes for the enzymes of different pathways namely purine metabolism; ubiquinone and another terpenoid-quinone biosynthesis; oxidative phosphorylation; pyrimidine metabolism; and thiamine metabolism. The name of enzymes and their respective pathways are as follows: (i) EC: 3.6.1.15—phosphatase enzyme involved in purine metabolism and thiamine metabolism pathways; (ii) EC: 1.6.5.2—dehydrogenase (quinone) enzyme of ubiquinone and other terpenoid-quinone biosynthesis pathways; (iii) EC:3.5.4.12—deaminase enzyme of pyrimidine metabolism pathway; and (iv) EC:7.1.1.2—reductase (H + -translocating) of oxidative phosphorylation pathway (Supplementary Figure 1).

### Bacterial challenge assay

**Figure 9:**
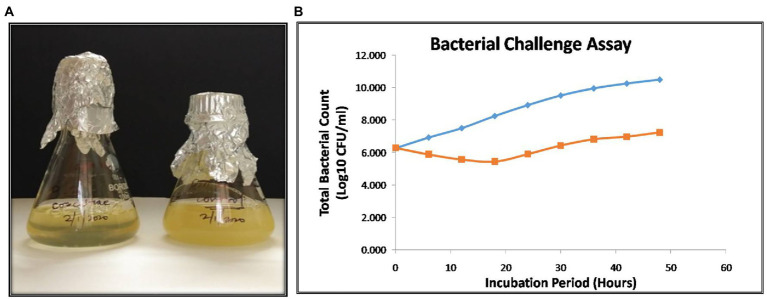
**(A)** Visible turbidity clearance on liquid culture media in phage infected culture as compared to control cultures after 48 h; **(B)** bacterial challenge assay of vB_XooS_NR08 on *Xanthomonas oryzae* pv*. oryzae* XooCG01 at 28°C. The phage NR08-treated (orange color line) bacterial count showed bacteriostasis up to 24 h as compared to bacterial control (blue color line); thereafter, the reduction in the count was 99.95% as compared to untreated bacterial control at 48 h.

The bacterial challenge assay of NR08 on its host bacteria was tested by evaluating the change in total colony count of host bacteria after NR08 infection at every 6-h interval up to 48 h. NR08 phage was effective against the host strain in the tested 0.1 MOI, and almost no bacterial growth was observed in the initial 24 h after co-inoculation. After 24 h, the minimal steady growth of XooCG01 occurred indicating that the equilibrium between cell growth and lysis has been achieved (Figure 9A). The phage NR08-treated bacterial count showed bacteriostasis up to 24 h; thereafter, up to the studied period of 48 h, there was more than 3 log_10_ reduction in the bacterial count, i.e., 99.95% reduction as compared to untreated bacterial control (Figure 9B). These results suggest that NR08 can be explored as a potent phage candidate for biocontrol of bacterial blight infection.

### Efficacy of Xoophage NR08 in control of BLB infection

**Figure 10:**
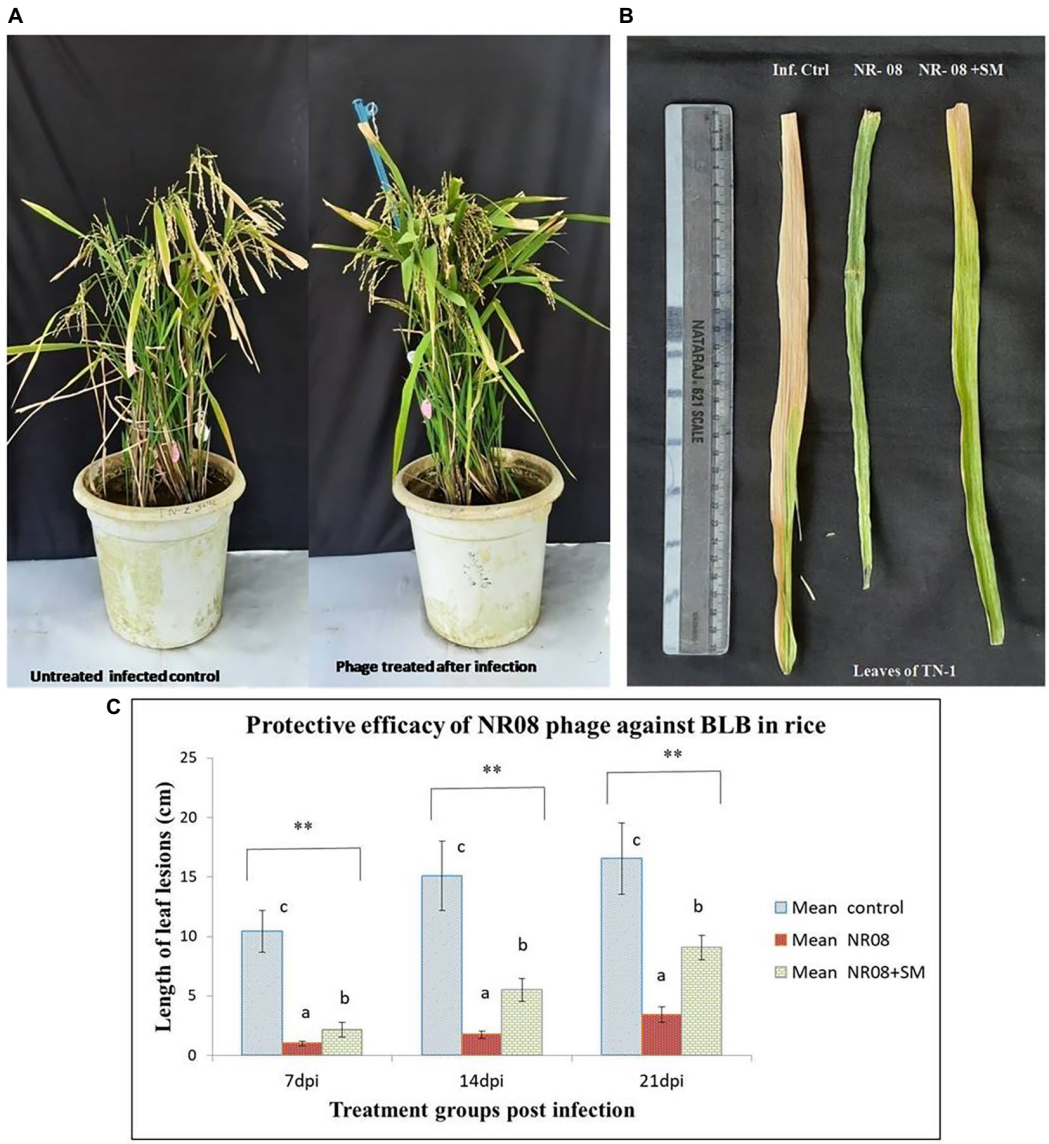
Protective efficacy of phage in rice pot against BB in rice. **(A)** Phage-treated TN-1 rice plants showing significant reduction of BLB symptoms as compared to untreated control plants after 14 days of infection and 11 days of phage treatment; **(B)** leaves of rice plants highly susceptible TN-1 variety after 15 days of infection with XooCG01 by leaf clip method and 11 days of phage treatment with NR08 phage in two preparations: neat phage (NR08); phage with 2% skim milk (NR08 + SM) by spray method. Infected control plants were infected with XooCG01 by leaf clip method and sprayed with sterile water only on the day of phage treatment; **(C)** Protective efficacy of NR08 phage-treated plants and NR08 along with 2% skim milk (SM) treated plants in comparison with untreated infected control plants of rice for bacterial blight symptoms in terms of length of leaf lesions (cm) at 7, 14, and 21 dpi. ** indicates a significant difference between all the three groups *p* < 0.01 at different points in time.

In the *in-planta* pot experiments for the effectiveness of selected phage against BLB pathogen, the treatment includes a single application of phage preparations 72 h after the bacterial inoculation, while infected untreated rice plants were used as control. Application of treatments was performed with a handheld sprayer during the evening hours of the day. Plants were assessed for disease severity on 7, 14, and 21 days post-infection or 4, 10, and 18 days post-treatment. Data sets of measured lesion lengths from 10 observations per group were used for analysis. Phage-treated rice plants showed a significant reduction of BLB symptoms as compared to untreated control plants (Figures 10A,B). Within the first 3 weeks of observation, the disease leaf lesion length was recorded as lowest in the NR08 phage treatment with a mean length of 1.02 ± 0.20 cm, 1.74 ± 0.32 cm, and 3.43 ± 0.63 cm at 7, 14, and 21 dpi, respectively, followed by the NR08 phage preparation supplemented with 2% skim milk showing leaf lesion length of 2.15 ± 0.62 cm, 5.53 ± 0.96 cm, and 9.08 ± 0.99 cm at 7, 14, and 21 dpi, respectively, and were significantly different at *p* < 0.01 from untreated infected control showing leaf lesions length of 10.45 ± 1.77 cm, 15.1 ± 2.91 cm, and 16.55 ± 3.02 cm at 7, 14, and 21 dpi, respectively (Table 4; Figure 10). The NR08 treatment provided the highest disease control efficacy ranging from 79.27% to 90.23% at different dpi followed by NR08 supplemented with SM treatment at 45.13% to 79.42% at different dpi, between which there was a significant difference. In both the experimental phage treatment groups, the application of phage NR08 treatment significantly reduced the lesion on rice leaves as compared to the untreated infected control (Table 4).

**Table 4:** Diseased mean leaf lesion length (cm) for the treatment groups of rice plants inoculated with NR08 phage preparation and BLB pathogen as observed up to 21 days post-infection.

Treatment groups	Mean average diseased leaf lesion length (cm) ± SD by XooCG01 at days post infection (dpi)			Disease suppression efficacy (%) over untreated control at dpi		
	7 dpi	14 dpi	21 dpi	7 dpi	14 dpi	21 dpi
Untreated infected control	10.45^c^ ± 1.77	15.1^c^ ± 2.91	16.55^c^ ± 3.02	**---**	**---**	**---**
Phage NR08 treated	1.02^a^ ± 0.20	1.74^a^ ± 0.32	3.43^a^ ± 0.63	90.23%	88.47%	79.27%
Phage NR08 with 2% skim milk treated	2.15^b^ ± 0.62	5.53^b^ ± 0.96	9.08^b^ ± 0.99	79.42%	63.37%	45.13%

Values with the different superscripts within the row are statistically significantly different using Duncan’s multiple range test at *p* < 0.01.

## Discussion

Bacterial blight (BLB) disease caused by *Xanthomonas oryzae* pv. *oryzae*, one of the earliest known diseases continues to be the most devastating disease in rice resulting in more than 50% yield losses. This disease also interferes with the maturation process affecting the grain quality (Lu et al., 2014). Nowadays as evident by recent publications, phage therapy has regained renewed and increased interest among researchers in agriculture and phage-based biocontrol research targets the most abundant and devastating plant pathogens (Buttimer et al., 2017). In recent years, phages have been isolated against bacteria infecting the majority of crops. Several studies showed that phage therapy using lytic bacteriophages was plausible for controlling plant pathogenic bacteria associated with several systems as they are cheap, self-amplifying, self-eliminating, and safe for the host organism (Korniienko et al., 2022). Due to ineffective chemical control methods, increasing antimicrobial resistance, and increasing environmental concerns, bacteriophages offer alternative management strategies for controlling plant diseases caused by bacterial pathogens. In this communication, we have isolated the bacteriophages specific to *X. oryzae* pv. *oryzae* and discussed about complete characterization of NR08 phage, i.e., its biological properties, survivability to environmental conditions, genomic characteristics, and efficacy of phage NR08 against *Xoo* in rice pots as a potent biocontrol agent to mitigate bacterial leaf blight.

We have isolated 19 lytic phages specific to *Xoo* from water, soil, and debris collected mostly from the infected rice field having an abundance of the host bacterium. Similarly, the majority of *Xanthomonas oryzae* phages were isolated from *Xoo*-infected leaves, infected paddy water, and soil (Nakayinga et al., 2021) indicating that the phages can be easily isolated from infected fields as they are obligate parasites of their host bacterium and can only survive in the environment having an abundance of their host. The host specificity of 19 Xoophages revealed that all 19 phages infected all five *Xoo* strains belonging to Chhattisgarh and Odisha states, while only 4 phages, *viz.*, NR08, NR12, NR15, and NR18, possess infectivity for *Xoo* strain from state Tripura. This might be due to variations in the binding capability of phages or change in receptor determinants for the host *Xoo*TR01 strain isolated from the northeastern state of India. Furthermore, NR08 has a broad range of *Xoo* host strains, as it infected all the six tested *Xoo* strains. None of the phages were able to infect the bacteria other than *Xoo* thus indicating their host specificity. They even do not possess any lytic activity for other species of genus *Xanthomonas*, i.e., *X. campestris* and, thus, are highly host-specific for their bactericidal activity and will not harm the beneficial microflora present in the soil. This minimizes the risk of phage attack on beneficial microbes present in the agricultural field, if in future used as a biocontrol agent against *Xoo*.

Morphological analysis, *via* TEM, showed all 19 phages belonging to order Caudovirales with typical head and tail morphology. Among these, 18 were classified as myoviruses (4), siphoviruses (12), and podoviruses (2) based on their tail characteristics, while we were unable to classify phage NR-14 to any of the phage family. NR14 phage had approximately 286 nm head width, 314 nm head length, 78 nm tail length, and 48 nm tail width, but its morphology does not resemble the families of *Myoviridae*, *Siphoviridae*, or *Podoviridae*. As per our limited knowledge, the morphology of NR-14 appears differently and hence presently could not be classified in any family. This limitation can be resolved through genome analysis of phage NR14 which will be communicated in our future publications. Similarly, previously reported Xoophages, namely, OP1, OP2, P4L, P43M, XP10, Xop411, Xoo-sp13, Xoo-sp15, Nɸ-1, and Nɸ-3, belong to families *Myoviridae*, *Siphoviridae*, and *Podoviridae* having genome as dsDNA with size exceeding 40 kb (Chae et al., 2014; Ogunyemi et al., 2019; Liu et al., 2021; Nazir et al., 2021). During the preparation of samples for electron microscopy, we employed both the filtered phage lysates and the PEG precipitation phage preparations. Fortunately, we were able to obtain sufficiently good micrographs using filtered phage lysates; hence, PEG precipitates were not used further as there may be chances of loss of phage properties during the PEG precipitation process (Carroll-Portillo et al., 2021). In this study, the complete characterization of the NR08 phage was done. The phage NR08, nomenclature as vB_XooS_NR08, isolated from rice field water, is a lytic phage producing a clear zone around the streaked lines on the bacterial lawn of different *Xoo* strains. NR08 belongs to *Siphoviridae* family with B1 morphotype, having an apparently icosahedral head of approximately 56.84 ± 2.58 nm head width, 58.72 ± 2.16 nm head length, and 156.42 ± 8.63 nm long non-contractile tail. NR08 phage produced clear plaques of 1–2 mm size and had a wide host range infecting all the six *Xoo* isolates. NR08 had a latent period of 40 min followed by a burst period between 40 and 70 min with a burst size of 250 virion per bacterial cell, whereas the previous report indicated that Xoo-sp2 has a latent period of approximately 3 h with a burst size of 350 virions per infected cell. Similarly, NR08 has a significantly shorter latent period and comparatively low burst size than that of other reported Xoo-sp2 phages (Dong et al., 2018).

Various environmental factors such as temperature, pH, and UV radiation inactivate phage particles by damaging their structural element (Gill and Abedon, 2003). As for thermal tolerance, the phage NR08 can remain nearly 100% viable at 4°C–40°C in the laboratory as well as in the field condition and with an increase in temperature beyond 50°C its viability reduces sharply. As for the pH stability, NR08 remains at least 80% viable in a pH range of 5–9, with an optimum pH of 6–8. Viability also reduces drastically within few minutes when the phage was exposed to UV light in the laminar flow hood. However, more than 75% of phage NR08 can remain viable for 135 min when exposed to direct sunlight. This feature of phage will be of advantage when applied in field conditions in India, as the environmental temperature during cropping season in most of the rice cultivating states in India rarely exceeds beyond 50°C. Furthermore, the tolerance of phage to direct sunlight is more than 2 h which is supposed to be sufficient enough time for invading and getting access to the plant tissues in the field when phage formulations containing skim milk, sucrose, or lactose along with phage will be applied. The inclusion of such substances in formulations has been shown to enhance the viability of phages in environmental conditions (Balogh et al., 2003). Furthermore, to overcome these limitations, strategies like applying the phages in the evening hours of the day to reduce exposure to sunlight and temperature can also be effective. Phage is inactivated by routine laboratory chemicals such as formalin, SDS, and aqueous phenol which may be a relevant biosafety aspect. After the application of phage in the field, the phage count increases if their target bacterial host species are accessible to them in the environment. Thus, even if their number is less due to any environmental constraints, the availability of their host during pathogen infection in the field, the phages can get easy access to their host and multiply to increase their number, infecting more and more bacterial cells. However, they tend to persist in high numbers in any environment only as long as their host is available. They are self-limiting in the absent of their host (Iriarte et al., 2012). Fujiwara et al. (2011) have identified phages that persist at relatively stable concentrations for several weeks in soil under favorable conditions.

The genome of the phage vB_XooS_NR08 is a linear double-stranded DNA (98,812 bp) with 52.9% G + C content that encodes 141 putative open reading frames (ORFs) and 1 tRNA^Gln^ gene. Among the predicted ORFs, 99 were predicted to have some specific functions, while 42 predicted ORFs (approximately 30%) were hypothetical having unknown functions. The functional ORFs of the genome were organized into four functional modules: DNA packaging module, phage structural and morphogenesis module, nucleotide metabolism module, and host lysis module. The morphogenesis module harbors genes encoding the phage capsid and tail structural component including chaperon protein, scaffold proteins, and others. The nucleotide metabolism module harbors genes related to phage DNA polymerase, DNA helicase, DNA ligase, phage exonuclease, and terminase. While DNA packaging module includes genes for DNA-binding proteins, phage portal proteins, phage terminase large and small subunits, DNA packaging proteins, and aminotransferases. Similarly, the host lysis module contains ORFs encoding for holins, lysozymes, Clp protease, phage spanin Rz, and peptidoglycan endopeptidase. The genome annotation results showed the absence of genes associated with integrase suggesting the lytic life style of phage NR08. It also showed the absence of genes associated with pathogenicity, virulence factors, antibiotic resistance, toxicity, and allergens. Thus eliminating the possibility of a lysogenic cycle through which the bacterial pathogenic islands (PAIs), virulence genes virulence factors, and AMR genes may get transferred to other bacteria *via* phages. Thus, suggesting this phage may be suitable for further biocontrol applications without harm to mankind and the environment (Yuan et al., 2019).

Enzymes of lytic mechanisms are used to lyse the infected host bacterium. In most of the previously reported Xoophages, members of holins and endolysins have been found involved in the lysis function. Holins create holes or pores in the lipid bilayer membrane of the host bacterium, thus allowing other enzymes of the lysis module to pass through the holes, whereas Rz spanin like lysis protein assists the lysozyme to enter the outer membrane from the inner membrane while one among the endolysins is peptidoglycan endopeptidase escapes through the holes and cleaves the peptide moiety, the structural portion of the bacterial cell wall (Wu et al., 2021). All these enzymatic activities lead to the difference in osmotic pressure between the bacterial cell and its surrounding environment, thus finally resulting in complete cell lysis and release of progeny phage particles. The ability of the bacteriophage and their gene products to inhibit growth or kill bacteria makes them potential antimicrobial sources. These enzymes are a promising alternative to antibiotics as they are essential components of the lytic life cycle of the phage (Abdelrahman et al., 2021). Phage NR08 is unique as it harbors all of these essential enzymes associated with lysis function, *viz.*, holins, lysozyme, peptidoglycan endopeptidase, and Rz sapnin protein, thus making this a novel phage with a more efficient lytic mechanism. Furthermore, the NR08 phage contains seven genes encoding HNH homing endonucleases, a very common family of protein generally associated with nuclease activity. The homing endonucleases are enzymes that stabilize the phage genetic information. HNH is involved in the initiation of the non-reciprocal transfer of DNA fragments containing their own genes and the flanking sequences by cleaving the recipient DNA (Brok-Volchanskaya et al., 2008). Similarly, multiple copies of HNH endonuclease genes are present in XP10, OP1, and XOP411 Xoophages. These enzymes are involved in the conservation of functionally important residues thus preserving their ability to bind DNA. Furthermore, through the process of gene translocation or duplication, these genes may populate the genomes. Their presence may also be involved in domain duplication of the tail fibers altering the host range (Lee et al., 2007).

Based on comparative bioinformatics analysis, the closest relatives of phage NR08 are *Xanthomonas* phage Samson (acc. no. MN062187), *Pseudomonas* phage PaMx42 (acc. no. JQ067092), and *Pseudomonas* phage Guyu (acc. no. MZ927746) with 40% query coverage sharing 93.74% to 96.68% identity (Clark et al., 2019; Loh et al., 2021). Comparative genomics of NR08 phage with ten Xoophages reported by Kovács et al. (2019) (Accession no. MG944227 to MG 944236) showed that the matched genome sizes were approximately 43–44 kb out of 98.8 kb genome size of NR08, whereas the average alignment percentage (AP) of NR08 with other 12 Xoophages ranged from 0.32 to 1.25. The genome size of NR08 (98.8 kb) is almost double of most of the previously reported phages (43–47 kb). This clearly indicates that the most of genome sequence information is novel for bacteriophage, vB_XooS_NR08, infecting *Xanthomonas oryzae* pv*. oryzae*. The phage was found to contain ORFs associated with virulence of phage leading to lytic cycle which is a most potential feature for the selection of phage as a biocontrol agent. NR08 phage showed low sequence similarity to other known phages representing a new addition as a novel phage to the Xoophage family. Its genome contains approximately 30% of not functionally annotated open reading frames, thus creating an interesting area to understand the biology and functioning of this novel phage.

In bacterial challenge assay, the phage NR08 showed bacteriostasis up to 24 h; thereafter, the reduction in the bacterial count was 99.95% as compared to untreated bacterial control in 48 h of incubation suggesting the potential of NR08 as a potent phage candidate for biocontrol of bacterial leaf blight infection. In pot assay, the single application of neat phage was more effective and statistically different from the phage preparation supplemented with 2% skim milk at every point of observation, i.e., 7, 14, and 21 dpi. Both the phage treatment variants/groups were statistically different from infected untreated control at 7, 14, and 21 dpi at *p* < 0.01. Furthermore, the disease suppression efficacy of neat phage treatment over the untreated infected control was 90.23%, 84.47%, and 79.27% at 7, 14, and 21 dpi, respectively. Similarly, in phage preparation added with 2% skim milk, the disease suppression efficacy over untreated control was 79.42%, 63.37%, and 45.13% at 7, 14, and 21 dpi, respectively. Thus, the efficacy of phage as a biological agent in the control of BLB was confirmed in pot experiments. There is a significant reduction in the intensity or severity of BLB disease compared to the untreated control. However, a single application of phage did not maintain a constant level of efficacy. Hence, suggesting the second or booster application of phage at few day intervals may be required to maintain or increase the efficacy of phage preparation. Similarly, in a study, Dong et al. (2018) reported that the length of the lesions on the rice leaves treated with Xoo-sp2 was significantly less (*t*-test, *p* < 0.01) than those in both control groups from day 9 post-infection. The length of the leaves lesions of rice on day 12 post-infection in the Xoosp2 treated group was 13.31 ± 1.69 cm, which was reduced by one-third in comparison with that in the two control groups treated with sterile water or skimmed milk, 20.83 ± 2.43 and 19.29 ± 2.07 cm, respectively. Similarly, the phage preparations were used to control various bacterial plant pathogens like *R. solanacearum* (Ramírez et al., 2020), *Xanthomonas* sp. (Tewfike and Desoky, 2015; Ibrahim et al., 2017), and *P. carotovorum sp. carotovorum* (Zaczek-Moczydłowska et al., 2020), and the phage preparations were found effective in limiting the disease by the researchers in their studies. Furthermore, it was evident from the observations that the addition of skim milk to phage preparation at 2% final concentration did not enhance the protective efficacy of phage as compared to neat phage application. The leaf lesion length of skim milk phage preparation is significantly more compared to the neat phage application suggesting that the addition of skim milk did not improve the protective efficacy of phages. Similarly, Balogh et al. (2008) also reported that the treatment with phage was ineffective if applied with a protective formulation containing skim milk. However, various studies have suggested that formulations containing skim milk, sucrose, dextrose, or tryptone can effectively protect the phage particles from the deleterious effect of UV light and other environmental factors associated with the survival of phage on field conditions (Balogh et al., 2003; Gašić et al., 2018). Furthermore, the studies suggested that high level of sugar and protein in skim milk has a beneficial effect on the survival of viruses (Iriarte et al., 2007). Avoiding daylight duration during the application or applying phage suspensions at the end of the light photoperiod and/or adding protective components in the bacteriophage preparations can improve phage-based biocontrol strategies. Balogh et al. (2003) demonstrated that applying phage to tomato leaves in the evening hours of the day resulted in longer phage persistence, giving phage more time to infect and kill their bacterial host.

In conclusion, this study describes the isolation, characterization, and application of vB_XooS_NR08, a novel bacteriophage against *Xoo*. It exhibits high specificity, broad *Xoo* host range, stability at a broad range of temperatures, and pH conditions and has a short latent period and large burst size. The morphological analysis indicated the NR08 phage as a member of the family *Siphoviridae*. Moreover, the genomic analysis indicated the NR08 phage has a lytic life cycle with strong lysis modules. It exhibited a strong ability to kill the *Xoo* pathogen both *in vitro* and *in vivo* conditions, suggesting this novel Xoophage may be an efficient, potent, and valuable candidate for the biological control of bacterial leaf blight of rice.

## Data availability statement

The datasets presented in this study can be found in online repositories. The names of the repository/repositories and accession number(s) can be found at: https://www.ncbi.nlm.nih.gov/genbank/, PRJNA844261; SRR19757545; and OQ055246.

## Author contributions

LJ isolated and characterized the phage, carried out the experiments, analyzed the data, and wrote the manuscript. VK performed bioinformatics analysis, carried out the experiments, analyzed the data, and wrote the manuscript. SJ collected samples and carried out plant experiments. PK and PG designed the experiment and revised the manuscript. All authors contributed to the article and approved the submitted version.

## Conflict of interest

The authors declare that the research was conducted in the absence of any commercial or financial relationships that could be construed as a potential conflict of interest.

## Publisher’s note

All claims expressed in this article are solely those of the authors and do not necessarily represent those of their affiliated organizations, or those of the publisher, the editors and the reviewers. Any product that may be evaluated in this article, or claim that may be made by its manufacturer, is not guaranteed or endorsed by the publisher.

## Footnotes

^1^https://phage.ai/

^2^http://stat.iasri.res.in/sscnarsportal/main.do
