# Supplementary material for: Isolation of bacteriophages infecting Xanthomonas oryzae pv. oryzae and genomic characterization of novel phage vB_XooS_NR08 for biocontrol of bacterial leaf blight of rice
Source: Front Microbiol. 2023 Mar 16;14:1084025. doi: 10.3389/fmicb.2023.1084025 (PMC10061587; doi:10.3389/fmicb.2023.1084025)
Supplement: Supplementary file 1 [file Data_Sheet_1.docx]

***Supplementary Material***

# Supplementary Figures and Tables

Supplementary Table 1: Annotated ORFs in the genome of phage NR08 which are classified based on their function in different modules.

| **Module type** | **Feature_id** | **Strand** | **Length** | **E-Value** | **Functions** |
| --- | --- | --- | --- | --- | --- |
| **Lysis module** | [fig\|2596676.14.peg.9](http://rast.nmpdr.org/seedviewer.cgi?page=Annotation&feature=fig\|2596676.14.peg.9) | + | 366 | 1.41E-69 | Phage holin |
|  | [fig\|2596676.14.peg.15](http://rast.nmpdr.org/seedviewer.cgi?page=Annotation&feature=fig\|2596676.14.peg.10) | + | 738 | 1.40E-174 | Clp protease ClpP |
|  | [fig\|2596676.14.peg.32](http://rast.nmpdr.org/seedviewer.cgi?page=Annotation&feature=fig\|2596676.14.peg.11) | + | 210 | 8.47E-40 | Phage holin |
|  | [fig\|2596676.14.peg.33](http://rast.nmpdr.org/seedviewer.cgi?page=Annotation&feature=fig\|2596676.14.peg.12) | + | 534 | 8.95E-100 | Phage lysine or lysozyme  (ACLAME 13) |
|  | [fig\|2596676.14.peg.34](http://rast.nmpdr.org/seedviewer.cgi?page=Annotation&feature=fig\|2596676.14.peg.13) | + | 297 | 6.75E-45 | Phage spanin Rz |
|  | [fig\|2596676.14.peg.91](http://rast.nmpdr.org/seedviewer.cgi?page=Annotation&feature=fig\|2596676.14.peg.14) | + | 315 | 1.95E-48 | holin |
|  | [fig\|2596676.14.peg.92](http://rast.nmpdr.org/seedviewer.cgi?page=Annotation&feature=fig\|2596676.14.peg.15) | + | 489 | 6.18E-85 | Phage protein---Rz |
|  | [fig\|2596676.14.peg.135](http://rast.nmpdr.org/seedviewer.cgi?page=Annotation&feature=fig\|2596676.14.peg.16) | + | 4716 | 0 | Phage peptidoglycan hydrolase |
| **Phage assembly and packaging** | [fig\|2596676.14.peg.140](http://rast.nmpdr.org/seedviewer.cgi?page=Annotation&feature=fig\|2596676.14.peg.18) | + | 135 |  | Phage head or portal protein (ACLAME 121) |
|  | [fig\|2596676.14.peg.12](http://rast.nmpdr.org/seedviewer.cgi?page=Annotation&feature=fig\|2596676.14.peg.19) | + | 159 | 3.43E-14 | Phage DNA binding protein |
|  | [fig\|2596676.14.peg.13](http://rast.nmpdr.org/seedviewer.cgi?page=Annotation&feature=fig\|2596676.14.peg.20) | + | 1686 | 0 | terminase DNA packaging protein A |
|  | [fig\|2596676.14.peg.14](http://rast.nmpdr.org/seedviewer.cgi?page=Annotation&feature=fig\|2596676.14.peg.21) | + | 1299 | 0 | Phage portal protein |
|  | [fig\|2596676.14.peg.17](http://rast.nmpdr.org/seedviewer.cgi?page=Annotation&feature=fig\|2596676.14.peg.22) | + | 348 | 2.91E-74 | Phage DNA packaging or aminotransferase (ACLAME 152) |
|  | [fig\|2596676.14.peg.36](http://rast.nmpdr.org/seedviewer.cgi?page=Annotation&feature=fig\|2596676.14.peg.23) | + | 117 | 1.65E-13 | phage terminase large subunit family protein |
|  | [fig\|2596676.14.peg.50](http://rast.nmpdr.org/seedviewer.cgi?page=Annotation&feature=fig\|2596676.14.peg.24) | - | 762 | 1.69E-92 | single-stranded DNA-binding protein |
|  | [fig\|2596676.14.peg.52](http://rast.nmpdr.org/seedviewer.cgi?page=Annotation&feature=fig\|2596676.14.peg.25) | - | 2241 | 0 | phage terminase large subunit family protein |
|  | [fig\|2596676.14.peg.76](http://rast.nmpdr.org/seedviewer.cgi?page=Annotation&feature=fig\|2596676.14.peg.26) | + | 375 | 1.09E-55 | putative portal protein |
|  | [fig\|2596676.14.peg.93](http://rast.nmpdr.org/seedviewer.cgi?page=Annotation&feature=fig\|2596676.14.peg.27) | + | 1614 | 0 | putative terminase small subunit |
|  | [fig\|2596676.14.peg.94](http://rast.nmpdr.org/seedviewer.cgi?page=Annotation&feature=fig\|2596676.14.peg.28) | + | 1515 | 0 | Phage terminase, large subunit |
|  | [fig\|2596676.14.peg.95](http://rast.nmpdr.org/seedviewer.cgi?page=Annotation&feature=fig\|2596676.14.peg.29) | + | 1095 | 0 | portal protein |
| **phage structural protein** | [fig\|2596676.14.peg.16](http://rast.nmpdr.org/seedviewer.cgi?page=Annotation&feature=fig\|2596676.14.peg.32) | + | 1173 | 0 | Phage major capsid protein |
|  | [fig\|2596676.14.peg.18](http://rast.nmpdr.org/seedviewer.cgi?page=Annotation&feature=fig\|2596676.14.peg.33) | + | 375 | 2.48E-80 | phage head closure protein |
|  | [fig\|2596676.14.peg.21](http://rast.nmpdr.org/seedviewer.cgi?page=Annotation&feature=fig\|2596676.14.peg.34) | + | 633 | 6.63E-123 | Phage tail fiber protein |
|  | [fig\|2596676.14.peg.25](http://rast.nmpdr.org/seedviewer.cgi?page=Annotation&feature=fig\|2596676.14.peg.35) | - | 252 | 1.04E-50 | major capsid protein |
|  | [fig\|2596676.14.peg.26](http://rast.nmpdr.org/seedviewer.cgi?page=Annotation&feature=fig\|2596676.14.peg.36) | + | 336 | 7.97E-53 | Phage tail, tail length tape-measure protein H |
|  | [fig\|2596676.14.peg.28](http://rast.nmpdr.org/seedviewer.cgi?page=Annotation&feature=fig\|2596676.14.peg.37) | - | 318 | 1.99E-42 | Phage tail fiber protein (ACLAME 365) |
|  | [fig\|2596676.14.peg.31](http://rast.nmpdr.org/seedviewer.cgi?page=Annotation&feature=fig\|2596676.14.peg.38) | + | 933 | 0 | Phage tail fiber protein (ACLAME 365) |
|  | [fig\|2596676.14.peg.45](http://rast.nmpdr.org/seedviewer.cgi?page=Annotation&feature=fig\|2596676.14.peg.39) | + | 2952 | 0 | central tail hub |
|  | [fig\|2596676.14.peg.65](http://rast.nmpdr.org/seedviewer.cgi?page=Annotation&feature=fig\|2596676.14.peg.40) | - | 252 | 3.03E-53 | Phage tail, tail length tape-measure protein H |
|  | [fig\|2596676.14.peg.98](http://rast.nmpdr.org/seedviewer.cgi?page=Annotation&feature=fig\|2596676.14.peg.41) | + | 429 | 6.25E-46 | major capsid protein |
|  | [fig\|2596676.14.peg.100](http://rast.nmpdr.org/seedviewer.cgi?page=Annotation&feature=fig\|2596676.14.peg.42) | + | 519 | 2.45E-122 | virion protein |
|  | [fig\|2596676.14.peg.101](http://rast.nmpdr.org/seedviewer.cgi?page=Annotation&feature=fig\|2596676.14.peg.43) | + | 381 | 7.71E-85 | virion structural protein |
|  | [fig\|2596676.14.peg.103](http://rast.nmpdr.org/seedviewer.cgi?page=Annotation&feature=fig\|2596676.14.peg.44) | + | 1542 | 0 | Possible peptidoglycan binding tail protein |
|  | [fig\|2596676.14.peg.104](http://rast.nmpdr.org/seedviewer.cgi?page=Annotation&feature=fig\|2596676.14.peg.45) | + | 429 | 3.87E-99 | major tail tube protein |
|  | [fig\|2596676.14.peg.108](http://rast.nmpdr.org/seedviewer.cgi?page=Annotation&feature=fig\|2596676.14.peg.46) | + | 963 | 0 | Phage tail tape measure |
|  | [fig\|2596676.14.peg.109](http://rast.nmpdr.org/seedviewer.cgi?page=Annotation&feature=fig\|2596676.14.peg.47) | + | 957 | 3.67E-155 | virion protein |
|  | [fig\|2596676.14.peg.110](http://rast.nmpdr.org/seedviewer.cgi?page=Annotation&feature=fig\|2596676.14.peg.48) | + | 1710 | 0 | virion structural protein |
|  | [fig\|2596676.14.peg.111](http://rast.nmpdr.org/seedviewer.cgi?page=Annotation&feature=fig\|2596676.14.peg.49) | + | 144 | 7.37E-24 | virion structural protein |
|  | [fig\|2596676.14.peg.112](http://rast.nmpdr.org/seedviewer.cgi?page=Annotation&feature=fig\|2596676.14.peg.50) | - | 195 | 2.49E-11 | virion structural protein |
|  | [fig\|2596676.14.peg.116](http://rast.nmpdr.org/seedviewer.cgi?page=Annotation&feature=fig\|2596676.14.rna.1) | - | 120 |  | Phage flagellar hook-length control protein fliK (ACLAME 758) |
|  | [fig\|2596676.14.peg.132](http://rast.nmpdr.org/seedviewer.cgi?page=Annotation&feature=fig\|2596676.14.peg.51) | + | 357 | 1.60E-77 | Phage tail length tape-measure protein 1 |
|  | [fig\|2596676.14.peg.133](http://rast.nmpdr.org/seedviewer.cgi?page=Annotation&feature=fig\|2596676.14.peg.52) | + | 459 | 1.90E-106 | conserved phage protein |
|  | [fig\|2596676.14.peg.136](http://rast.nmpdr.org/seedviewer.cgi?page=Annotation&feature=fig\|2596676.14.peg.53) | + | 378 | 6.76E-55 | Phage tail fiber protein |
| **Phage Morphogenesis module** | [fig\|2596676.14.peg.19](http://rast.nmpdr.org/seedviewer.cgi?page=Annotation&feature=fig\|2596676.14.peg.55) | + | 474 | 9.86E-108 | Phage capsid and scaffold |
|  | [fig\|2596676.14.peg.22](http://rast.nmpdr.org/seedviewer.cgi?page=Annotation&feature=fig\|2596676.14.peg.56) | + | 303 | 5.63E-64 | phage tail assembly chaperone |
|  | [fig\|2596676.14.peg.96](http://rast.nmpdr.org/seedviewer.cgi?page=Annotation&feature=fig\|2596676.14.peg.57) | + | 711 | 1.72E-158 | F-like head morphogenesis protein |
|  | [fig\|2596676.14.peg.97](http://rast.nmpdr.org/seedviewer.cgi?page=Annotation&feature=fig\|2596676.14.peg.58) | + | 975 | 0 | putative scaffold protein |
|  | [fig\|2596676.14.peg.102](http://rast.nmpdr.org/seedviewer.cgi?page=Annotation&feature=fig\|2596676.14.peg.59) | + | 456 | 8.82E-107 | head-tail joining protein |
|  | [fig\|2596676.14.peg.105](http://rast.nmpdr.org/seedviewer.cgi?page=Annotation&feature=fig\|2596676.14.peg.60) | + | 303 | 2.56E-67 | tail chaperonin |
|  | [fig\|2596676.14.peg.106](http://rast.nmpdr.org/seedviewer.cgi?page=Annotation&feature=fig\|2596676.14.peg.61) | + | 435 | 5.18E-99 | tail chaperonin |
|  | [fig\|2596676.14.peg.107](http://rast.nmpdr.org/seedviewer.cgi?page=Annotation&feature=fig\|2596676.14.peg.62) | + | 3828 | 0 | putative tail completion protein |
| **Phage Endonuclease** | [fig\|2596676.14.peg.29](http://rast.nmpdr.org/seedviewer.cgi?page=Annotation&feature=fig\|2596676.14.peg.64) | + | 120 | 1.81E-13 | endonuclease-like protein |
|  | [fig\|2596676.14.peg.30](http://rast.nmpdr.org/seedviewer.cgi?page=Annotation&feature=fig\|2596676.14.peg.65) | - | 333 | 1.04E-66 | endonuclease-like protein |
|  | [fig\|2596676.14.peg.57](http://rast.nmpdr.org/seedviewer.cgi?page=Annotation&feature=fig\|2596676.14.peg.66) | - | 930 | 0 | DNA endonuclease VII |
|  | [fig\|2596676.14.peg.79](http://rast.nmpdr.org/seedviewer.cgi?page=Annotation&feature=fig\|2596676.14.peg.67) | + | 345 | 1.06E-39 | Vsr endonuclease |
|  | [fig\|2596676.14.peg.131](http://rast.nmpdr.org/seedviewer.cgi?page=Annotation&feature=fig\|2596676.14.peg.68) | + | 3003 | 0 | endonuclease-like protein |
|  | [fig\|2596676.14.peg.6](http://rast.nmpdr.org/seedviewer.cgi?page=Annotation&feature=fig\|2596676.14.peg.6) | + | 516 | 3.75E-45 | Phage HNH homing endonuclease (ACLAME 27) |
|  | [fig\|2596676.14.peg.7](http://rast.nmpdr.org/seedviewer.cgi?page=Annotation&feature=fig\|2596676.14.peg.7) | + | 291 | 2.77E-11 | HNH endonuclease |
|  | [fig\|2596676.14.peg.35](http://rast.nmpdr.org/seedviewer.cgi?page=Annotation&feature=fig\|2596676.14.peg.8) | - | 432 | 2.80E-49 | Phage HNH homing endonuclease (ACLAME 27) |
|  | [fig\|2596676.14.peg.61](http://rast.nmpdr.org/seedviewer.cgi?page=Annotation&feature=fig\|2596676.14.peg.9) | - | 1692 | 0 | HNH endonuclease |
|  | [fig\|2596676.14.peg.115](http://rast.nmpdr.org/seedviewer.cgi?page=Annotation&feature=fig\|2596676.14.peg.10) | - | 678 | 3.44E-67 | Phage HNH homing endonuclease (ACLAME 27) |
|  | [fig\|2596676.14.peg.130](http://rast.nmpdr.org/seedviewer.cgi?page=Annotation&feature=fig\|2596676.14.peg.11) | + | 729 | 1.32E-161 | Phage HNH homing endonuclease (ACLAME 27) |
|  | [fig\|2596676.14.peg.138](http://rast.nmpdr.org/seedviewer.cgi?page=Annotation&feature=fig\|2596676.14.peg.12) | - | 174 |  | Phage HNH homing endonuclease (ACLAME 27) |
| **Phage metabolism module** | fig\|2596676.14.peg.4 | - | 93 | 2.45E-12 | transposase |
|  | fig\|2596676.14.peg.5 | + | 198 | 1.89E-40 | Mobile element protein |
|  | fig\|2596676.14.peg.38 | - | 474 | 6.43E-114 | Class A beta-lactamase (EC 3.5.2.6) => TEM family |
|  | fig\|2596676.14.peg.39 | - | 285 | 2.56E-63 | Mobile element protein |
|  | fig\|2596676.14.peg.40 | - | 165 | 7.29E-29 | ribosomal protein S8 |
|  | fig\|2596676.14.peg.41 | + | 168 | 7.20E-32 | NADH dehydrogenase subunit F |
|  | fig\|2596676.14.peg.42 | + | 549 | 5.01E-130 | Phage ea22 protein |
|  | fig\|2596676.14.peg.43 | - | 189 | 2.11E-39 | RNA polymerase-associated protein RapA |
|  | fig\|2596676.14.peg.44 | - | 189 | 4.17E-39 | RNA polymerase-associated protein RapA |
|  | [fig\|2596676.14.peg.46](http://rast.nmpdr.org/seedviewer.cgi?page=Annotation&feature=fig\|2596676.14.peg.45) | - | 2073 | 0 | Phage DNA polymerase |
|  | [fig\|2596676.14.peg.47](http://rast.nmpdr.org/seedviewer.cgi?page=Annotation&feature=fig\|2596676.14.peg.46) | - | 1164 | 0 | replicative clamp |
|  | [fig\|2596676.14.peg.48](http://rast.nmpdr.org/seedviewer.cgi?page=Annotation&feature=fig\|2596676.14.peg.47) | - | 1656 | 0 | DNA helicase, phage associated; Type III restriction enzme |
|  | [fig\|2596676.14.peg.49](http://rast.nmpdr.org/seedviewer.cgi?page=Annotation&feature=fig\|2596676.14.peg.48) | - | 1104 | 0 | RecB exonuclease |
|  | [fig\|2596676.14.peg.51](http://rast.nmpdr.org/seedviewer.cgi?page=Annotation&feature=fig\|2596676.14.peg.49) | - | 159 | 1.59E-29 | recombinase |
|  | [fig\|2596676.14.peg.53](http://rast.nmpdr.org/seedviewer.cgi?page=Annotation&feature=fig\|2596676.14.peg.50) | - | 363 | 2.80E-59 | Phage DNA-directed RNA polymerase (EC 2.7.7.6) |
|  | [fig\|2596676.14.peg.56](http://rast.nmpdr.org/seedviewer.cgi?page=Annotation&feature=fig\|2596676.14.peg.56) | - | 408 | 3.19E-76 | Phage exonuclease |
|  | [fig\|2596676.14.peg.58](http://rast.nmpdr.org/seedviewer.cgi?page=Annotation&feature=fig\|2596676.14.peg.57) | - | 798 | 0 | Phage exonuclease |
|  | [fig\|2596676.14.peg.60](http://rast.nmpdr.org/seedviewer.cgi?page=Annotation&feature=fig\|2596676.14.peg.58) | - | 513 | 9.51E-21 | DNA polymerase I (EC 2.7.7.7), phage-associated |
|  | [fig\|2596676.14.peg.62](http://rast.nmpdr.org/seedviewer.cgi?page=Annotation&feature=fig\|2596676.14.peg.59) | - | 1317 | 0 | DNA polymerase I (EC 2.7.7.7), phage-associated |
|  | [fig\|2596676.14.peg.63](http://rast.nmpdr.org/seedviewer.cgi?page=Annotation&feature=fig\|2596676.14.peg.60) | - | 843 | 8.08E-169 | DNA helicase, phage-associated |
|  | [fig\|2596676.14.peg.64](http://rast.nmpdr.org/seedviewer.cgi?page=Annotation&feature=fig\|2596676.14.peg.61) | - | 318 | 2.74E-68 | DNA primase/helicase, phage-associated |
|  | [fig\|2596676.14.peg.69](http://rast.nmpdr.org/seedviewer.cgi?page=Annotation&feature=fig\|2596676.14.peg.62) | - | 210 | 9.27E-42 | putative nucleotide pyrophosphohydrolase |
|  | [fig\|2596676.14.peg.70](http://rast.nmpdr.org/seedviewer.cgi?page=Annotation&feature=fig\|2596676.14.peg.63) | + | 2316 | 0 | helix-turn-helix domain-containing protein |
|  | [fig\|2596676.14.peg.71](http://rast.nmpdr.org/seedviewer.cgi?page=Annotation&feature=fig\|2596676.14.peg.64) | + | 513 | 6.07E-94 | Phage DNA primase/helicase |
|  | [fig\|2596676.14.peg.72](http://rast.nmpdr.org/seedviewer.cgi?page=Annotation&feature=fig\|2596676.14.peg.65) | + | 270 | 1.93E-44 | peptide chain release factor 1 |
|  | [fig\|2596676.14.peg.86](http://rast.nmpdr.org/seedviewer.cgi?page=Annotation&feature=fig\|2596676.14.peg.66) | + | 375 | 7.57E-81 | Deoxycytidylate deaminase (EC 3.5.4.12) |
|  | [fig\|2596676.14.peg.88](http://rast.nmpdr.org/seedviewer.cgi?page=Annotation&feature=fig\|2596676.14.peg.67) | + | 576 | 2.65E-101 | TPA: MAG TPA: Nematode cuticle collagen N-terminal domain |
|  | [fig\|2596676.14.peg.99](http://rast.nmpdr.org/seedviewer.cgi?page=Annotation&feature=fig\|2596676.14.peg.68) | + | 375 | 1.14E-69 | DNA polymerase III subunits gamma and tau |
|  | [fig\|2596676.14.peg.114](http://rast.nmpdr.org/seedviewer.cgi?page=Annotation&feature=fig\|2596676.14.peg.69) | + | 267 | 1.38E-30 | DNA ligase, phage-associated |
|  | [fig\|2596676.14.peg.119](http://rast.nmpdr.org/seedviewer.cgi?page=Annotation&feature=fig\|2596676.14.peg.70) | - | 198 | 2.49E-27 | Phage inhibitor of transcription initiation and antiterminator |
|  | [fig\|2596676.14.peg.124](http://rast.nmpdr.org/seedviewer.cgi?page=Annotation&feature=fig\|2596676.14.peg.71) | - | 183 | 0 | Phage peptidase (ACLAME 1043) |
|  | [fig\|2596676.14.peg.137](http://rast.nmpdr.org/seedviewer.cgi?page=Annotation&feature=fig\|2596676.14.peg.137) | - | 420 | 6.40E-54 | putative secreted protein |
| **Domain of Unknown function (DUF)** | [fig\|2596676.14.peg.134](http://rast.nmpdr.org/seedviewer.cgi?page=Annotation&feature=fig\|2596676.14.peg.119) | + | 393 | 4.87E-91 | DUF1833 family protein |
|  | [fig\|2596676.14.peg.20](http://rast.nmpdr.org/seedviewer.cgi?page=Annotation&feature=fig\|2596676.14.peg.120) | + | 357 | 5.59E-77 | DUF3168 domain-containing protein |
|  | [fig\|2596676.14.peg.23](http://rast.nmpdr.org/seedviewer.cgi?page=Annotation&feature=fig\|2596676.14.peg.121) | + | 129 | 1.18E-17 | DUF1799 domain-containing protein |
|  | [fig\|2596676.14.peg.54](http://rast.nmpdr.org/seedviewer.cgi?page=Annotation&feature=fig\|2596676.14.peg.122) | - | 147 | 2.82E-23 | DUF5664 domain-containing protein |
| **tRNA** | [fig\|2596676.14.rna.1](http://rast.nmpdr.org/seedviewer.cgi?page=Annotation&feature=fig\|2596676.14.rna.1) | - | 735 | 1.08E-173 | tRNA-Gln-TTG |


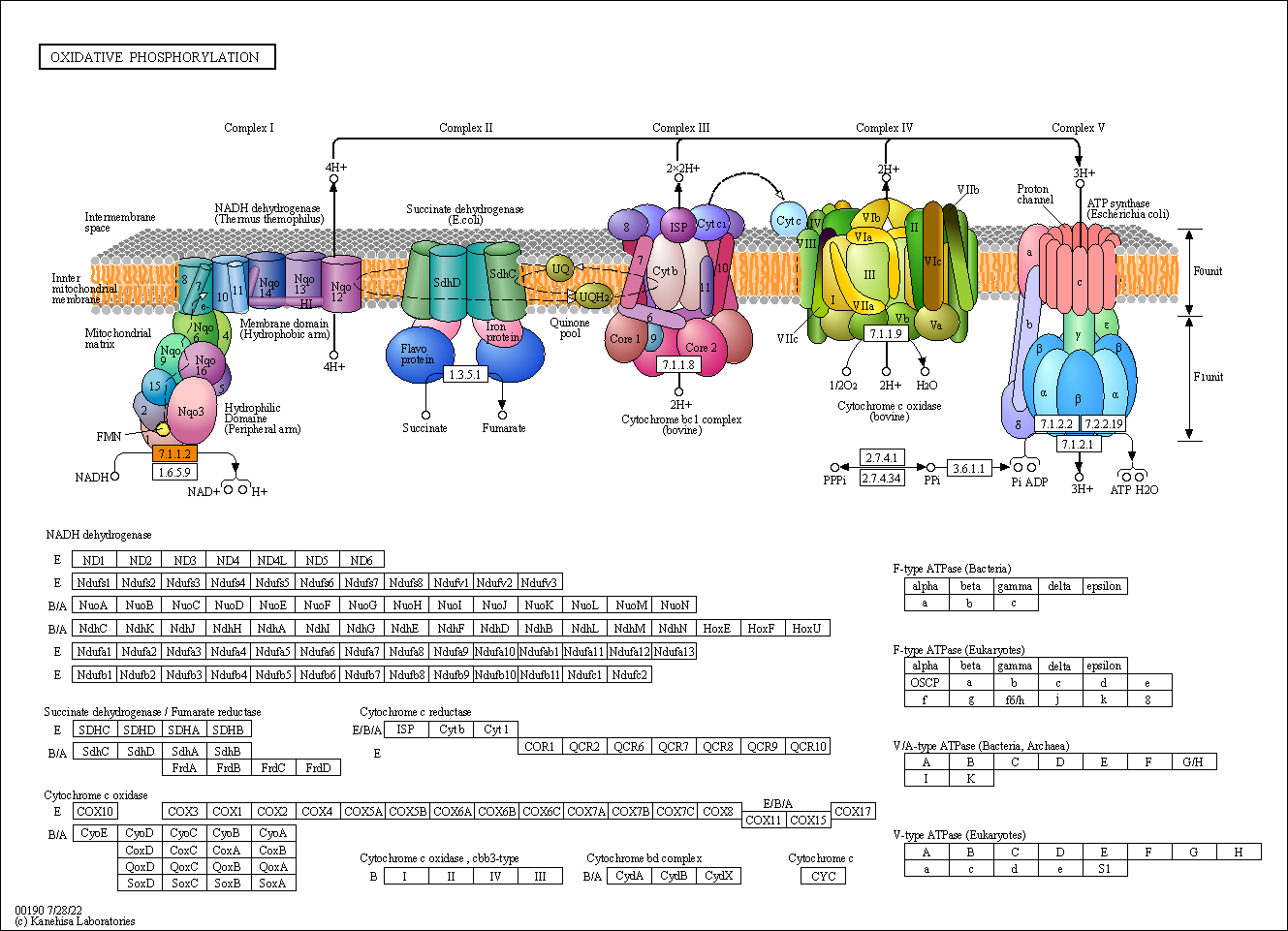


Supplementary Figure 1: Diagrammatic representation of KEGG pathway of NR08 phage showing the EC: 7.1.1.2 - reductase (H+-translocating) of Oxidative phosphorylation pathway
